# sp^2^-Iminosugars targeting human lysosomal β-hexosaminidase as pharmacological chaperone candidates for late-onset Tay-Sachs disease

**DOI:** 10.1080/14756366.2022.2073444

**Published:** 2022-05-16

**Authors:** Manuel González-Cuesta, Irene Herrera-González, M. Isabel García-Moreno, Roger A. Ashmus, David J. Vocadlo, José M. García Fernández, Eiji Nanba, Katsumi Higaki, Carmen Ortiz Mellet

**Affiliations:** a Department of Organic Chemistry, Faculty of Chemistry, University of Seville, Sevilla, Spain; b Department of Chemistry and Department of Molecular Biology and Biochemistry, Simon Fraser University, Burnaby, Canada; c Instituto de Investigaciones Químicas (IIQ), Consejo Superior de Investigaciones Científicas (CSIC) - Universidad de Sevilla, Sevilla, Spain; d Organization for Research Initiative and Promotion, Tottori University, Yonago, Japan

**Keywords:** Iminosugar, pharmacological chaperone, thiourea, thiazolidine, Tay-Sachs

## Abstract

The late-onset form of Tay-Sachs disease displays when the activity levels of human β-hexosaminidase A (HexA) fall below 10% of normal, due to mutations that destabilise the native folded form of the enzyme and impair its trafficking to the lysosome. Competitive inhibitors of HexA can rescue disease-causative mutant HexA, bearing potential as pharmacological chaperones, but often also inhibit the enzyme O-glucosaminidase (GlcNAcase; OGA), a serious drawback for translation into the clinic. We have designed sp^2^-iminosugar glycomimetics related to GalNAc that feature a neutral piperidine-derived thiourea or a basic piperidine-thiazolidine bicyclic core and behave as selective nanomolar competitive inhibitors of human Hex A at pH 7 with a ten-fold lower inhibitory potency at pH 5, a good indication for pharmacological chaperoning. They increased the levels of lysosomal HexA activity in Tay-Sachs patient fibroblasts having the G269S mutation, the highest prevalent in late-onset Tay-Sachs disease.

## 1. Introduction

*N*-Acetyl-β-hexosaminidase (Hex; EC 3.2.1.52) is a member of the glycosyl hydrolase family 20 (GH20) that catalyses the removal of terminal, non-reducing *N*-acetyl-β-d-glucosamine (GlcNAc) or galactosamine (GalNAc) residues from gangliosides, glycoproteins or glycosaminoglycans^1^. In humans two Hex isoforms are readily detectable, namely HexA and HexB. The first one is a heterodimer formed by α and β subunits, encoded respectively by the evolutionary related *HEXA* and *HEXB* genes, whereas the second one is the ββ homodimer^2^. The thermodynamically less stable αα homodimer (HexS) is also formed, but only reaches measurable levels when the β subunit is deficient. Although the α and β subunits possess independent active sites, dimerisation is a prerequisite for their in vivo biological function. Exclusively the α-subunit of HexA can hydrolyse the G_M2_ ganglioside (GM2), an intermediate in the biosynthesis and degradation of higher brain gangliosides, in lysosomes by specifically interacting with the G_M2_ activator protein (GM2AP) co-factor^3^. Disabling mutations in *HEXA*, *HEXB* or the gene encoding for GM2AP results in Tay-Sachs disease (TSD; OMIM #272800), Sandhoff disease (SD; OMIM #268800) or the less common AB variant (OMIM #272750), respectively, a subset of lysosomal storage disorders (LSDs) collectively referred to as G_M2_ gangliosidosis^4^. All the three are autosomal recessive conditions associated with phenotypic neurodegeneration and devastating consequences. Currently, there are no effective treatment options for any of these diseases^5^.

TSD is the best characterised of the G_M2_ gangliosidosis, with over 100 *HEXA* gene mutations categorised (http://www.hgmd.cf.ac.uk/). The frequency of asymptomatic heterozygotes is 1 in 300 live births in the general population (1 in 30 among Ashkenazy Jews), with a predicted frequency of 1 in 360,000 for homozygotes (1 in 2,900 among Ashkenazy Jews). Different genotypes result in different clinical phenotypes (infantile, juvenile or adult/chronic), with severity generally associating with the level of residual HexA activity permitted by different mutations (<0.5% of normal activity for infantile; 2%–5% for late on-set forms)^6^^,^^7^. Based on genotype/phenotype correlations, it has been suggested that 10% of the normal activity could be sufficient to prevent development of clinical symptoms^8^. Administration of recombinant HexA (enzyme replacement therapy; ERT) is unlikely to be clinically effective due to the inaccessibility imposed by the blood-brain barrier. Gene therapy using viral vectors has shown some promise in animal models^9^^,^^10^, but toxicity issues unveiled in non-human primates and low effectivity in clinical trials may seriously thwart translation to hospital settings^11^^,^^12^. Other approaches, such as the use of gene editing tools or brain permeable inhibitors of glycosphingolipid biosynthesis are under investigation^13^^,^^14^. Since many of the TSD-causative mutations do not compromise the catalytic site, but target the α chain of HexA to endoplasmic reticulum-associate degradation (ERAD), the development of pharmacological chaperones (PCs) that can stabilise the native folding of the protein despite its anomalous conformation and restore activity appears attractive^15^. Typically, a PC is a small molecule able to bind to the mutant enzyme at the ER, promote the correct folding and restore trafficking to the Golgi apparatus for maturation and then to the final destination. With few exceptions^16–18^, most reported PCs developed for LSDs are competitive inhibitors of the target enzyme; they however exert an effector action by dissociating from the corresponding mature enzyme: inhibitor complex in the presence of an excess of substrate in the lysosomes of patient cells^19–21^.

**Figure 1.**
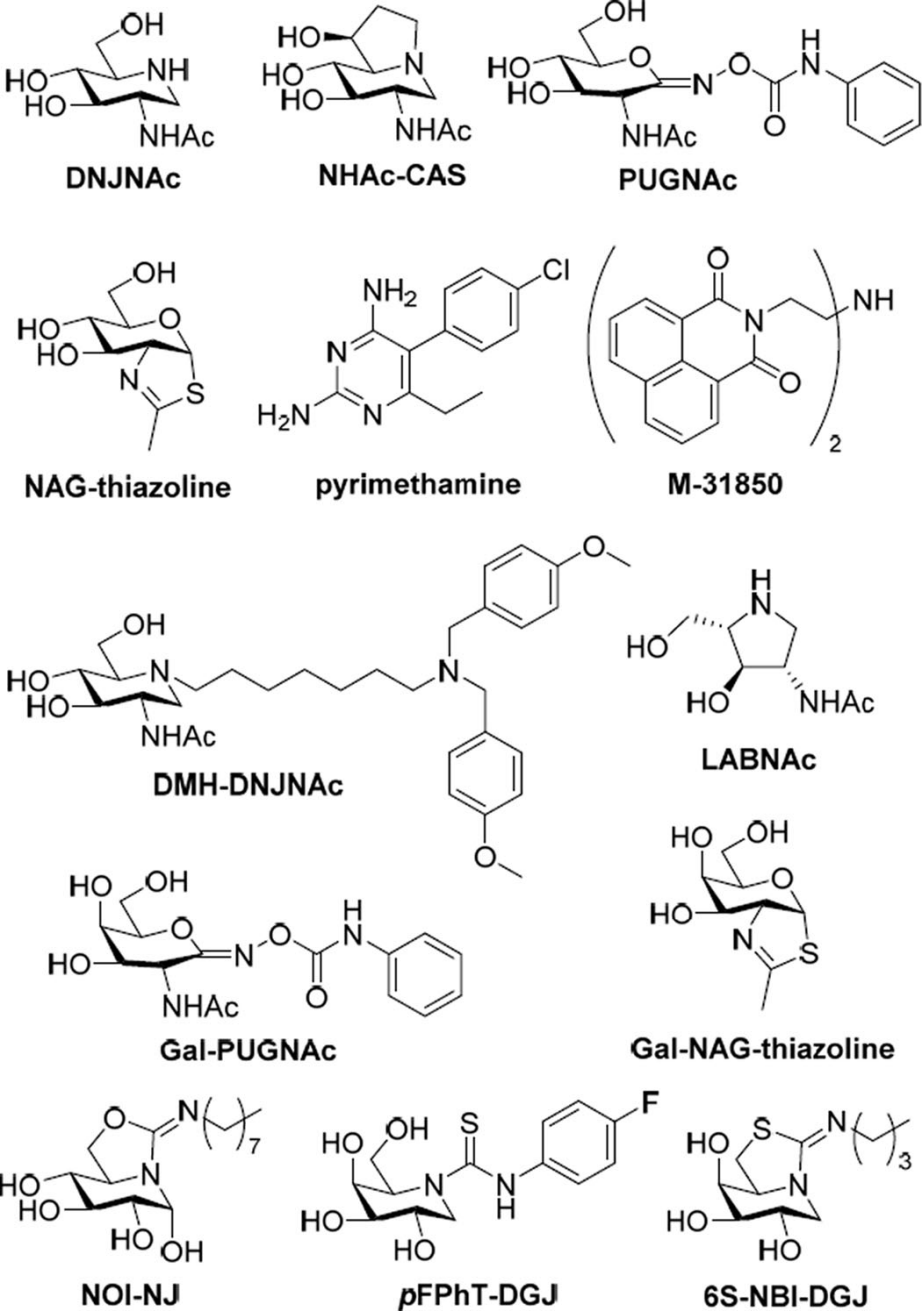
Structures of the HexA inhibitors DNJNAc, NHAc-CAS, PUGNAc, NAG-thiazoline, pymetahmine, M31850, DMH-DNJNAc, LABNAc, Gal-PUGNAc and Gal-NAG-thiazoline, and of the sp^2^-iminosugars NOI-NJ, pFPhT-DGJ and 6S-NBI-DGJ.

The prospective hexosaminidase PC candidates reported up to now include carbohydrate-related (glycomimetic) derivatives, among which 2-acetamido-1,2-dideoxynojirimycin (DNJNAc), 6-acetamido-6-deoxycastanospermine (NHAc-CAS), *O*-(2-acetamido-2-deoxy-d-glucopyranosylidene)amino *N*-phenylcarbamate (PUGNAc) and *N*-acetylglucosamine-thiazoline (NAG-thiazoline) are representative examples^22–26^, and noncarbohydrate-based compounds identified after high throughput screening, such as pyrimethamine or the naphtalimide derivative M-31850 (Figure 1)^27–30^. A main problem is that oftentimes those candidates are not hexosaminidase function-specific and also inhibit a similar glycosidase, O-GlcNAcase (OGA), a GH84 enzyme that hydrolyses GlcNAc residues from O-linked glycoproteins^31–33^. Three different strategies have been envisioned to address this issue: (a) installing substituents on the PC that can provide nonglycone interactions with the enzyme, as it is the case for *N*-[di(*p*-methoxybenzyl)aminoheptyl-NHAc-DNJ (DMH-DNJNAc)^34^; (b) exploiting five-membered instead of six-membered iminocyclitol (iminosugar) scaffolds (e.g. 2-acetamido-1,4-diamino-1,2,4-trideoxy-l-arabinitol; LABNAc)^35^^,^^36^; or (c) capitalising on GalNAc-related glycomimetic derivatives, such as the galactose-like epimers of PUGNAc and NAG-thiazoline (Gal-PUGNAc and Gal-NAG-thiazoline, respectively), since HexA but not OGA can accommodate GalNAc ligands in the active site (Figure 1)^37^^,^^38^. Whereas all three approaches have produced selective HexA inhibitors, their application as TSD-associated mutant HexA enhancers remains to be demonstrated. Up to now, pyrimethamine has been the only PC candidate assayed in TSD patients; unfortunately, the results were not satisfactory enough, highlighting the urgent need for developing more efficient and selective PC prototypes^39^.

**Figure 2.**
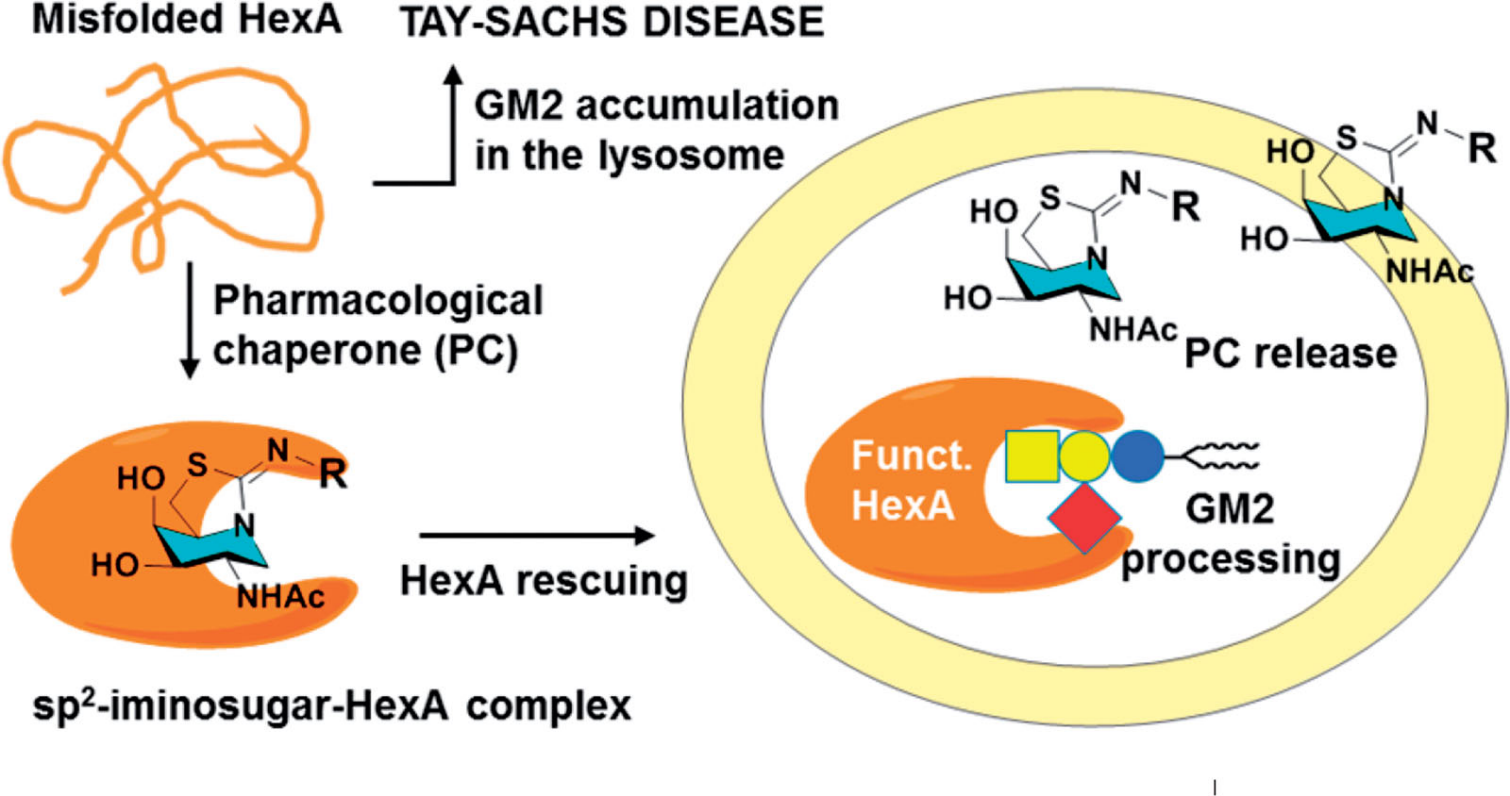
Schematic representation of the proposed pharmacological chaperone strategy tackling TSD causative misfolded HexA, based on active-site directed sp^2^-iminosugars (a generic bicyclic amphiphilic structure with a hydrophobic R substituent is depicted).

We and others have previously shown that replacing the amine-type endocyclic nitrogen atom in iminosugar frameworks by a pseudoamide-type nitrogen (sp^2^-iminosugars) represents a versatile strategy to achieve highly selective glycosidase ligands for fundamental studies on enzyme mechanisms^40–43^ and drug discovery, with applications ranging from cancer^44–46^ and inflammation^47^ to antiparasitic agents^48^. sp^2^-iminosugars are very well adapted to molecular diversity schemes, including modifications in the configurational pattern, the heterocycle framework and the nature of substituents^49–53^. Their outstanding chemical flexibility is ideally suited for structure-activity relationship studies, which has allowed optimising candidates capable of restoring the correct folding and trafficking of several LSD-causative mutant glycosidases. Current examples include β-glucocerebrosidase (e.g. 5 *N*,6*O*-[*N’*-octyliminomethylidene]nojirimycin; NOI-NJ)^54–56^, α-galactosidase (e.g. *N*-[*N’*-*p*-flurophenythiocarbamoyl]-1-deoxygalactonojirimycin; pFPhT-DGJ)^57^^,^^58^, β-galactosidase (e.g. 5 *N*,6*S*-[*N’*-butyliminomethylidene]-1-deoxy-6-thiogalactonojirimycin; 6S-NBI-DGJ)^59^^,^^60^, and α-mannosidase (e.g. 6-[*tert*-butoxycarbonylamino]hexyl 5 *N*,6*O*-[oxamethylidene]mannonojirimycin-1-yl amine; BocNHex-N-OMJ)^61^ in fibroblasts from patients suffering of Gaucher disease, Fabry disease, G_M1_ gangliosidosis and α-mannosidosis, respectively (Figure 1). Most interestingly, amphiphilic sp^2^-iminosugars exhibited very favourable chaperoning/inhibitory balances in patient-derived neurons^54^ and were shown to cross the blood-brain barrier in a murine model^60^, supporting their potential to prevent or slow neurological decline. Devising sp^2^-iminosugars with strong affinity and selectivity towards HexA represents, thus, an appealing tactic towards a PC therapy option for TSD^62^^,^^63^ (Figure 2). To probe this hypothesis, we have now synthesised a series of monocyclic and bicyclic GalNAc mimetics belonging to the sp^2^-iminosugar family bearing varying substituents, determined the inhibitory profile against different glycosidases and evaluated the hexosaminidase-enhancing capabilities in fibroblasts from healthy donors and TSD patients. The results provide a proof of concept of the potential of sp^2^-iminosugar-based PCs for the treatment of the late-onset form of TSD disease.

## 2. Results and discussion

### 2.1. Design rational and synthesis

**Scheme 1.**
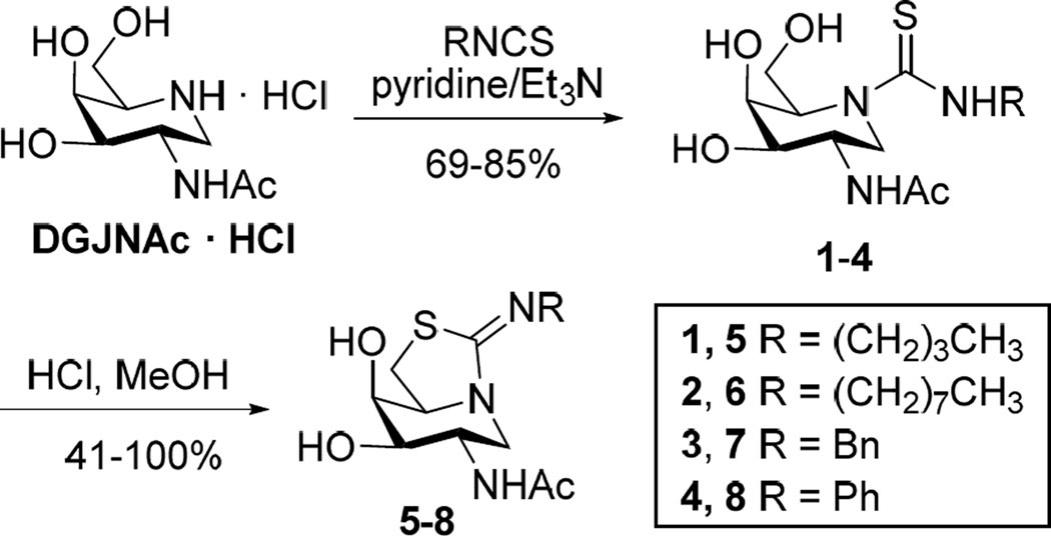
Synthesis of the DGJNAc thioureas **1**–**4** and the corresponding bicyclic isothioureas **5**–**8**.

We advanced that sp^2^-iminosugar glycomimetics featuring a substitution profile of stereochemical complementarity to GalNAc would discriminate the lysosomal *N*-acetyl-β-hexosaminidase from OGA, in view of their respective substrate specificities. The recent implementation of a scalable preparative method for accessing 2-acetamido-1,2-dideoxygalactonojirimycin (DGJNAc)^64^, as the corresponding hydrochloride, enabled the direct synthesis of the corresponding thiourea adducts **1**–**4** by coupling reactions with butyl, octyl, benzyl or phenyl isothiocyanate. Triethylamine was used as a base to promote deprotonation of the starting iminosugar salt. The transformations proceeded with total chemoselectivity and good yields, avoiding hydroxyl protection/deprotection steps, in agreement with the categorisation of the thiourea-forming reaction as a click-type conjugation method very well-suited for molecular diversity oriented schemes (Scheme 1)^65–69^. The resulting hydroxythioureas served at their turn as precursors for the bicyclic derivatives **5**–**8**. Intramolecular nucleophilic displacement of the primary hydroxyl group by the thiocarbonyl sulphur atom proceeded spontaneously in methanol at room temperature in the presence of hydrochloric acid, affording the target isothiourea-type sp^2^-iminosugars^70^ in yields that ranged from virtually quantitative in the case of *N’*-alkyl derivatives (**5**–**7**) to moderate (41%) for the *N’*-phenyl counterpart (**8**; Scheme 1).

### 2.2. Glycosidase inhibition properties against commercial enzymes

Compounds **1**–**8** were preliminary assayed for their glycosidase inhibitory properties against a panel of commercial glycosidases covering a variety of configurational and anomeric specificities, including bovine and almond β-glucosidase, yeast α-glucosidase and isomaltase, *Aspergillus niger* amyloglucosidase, *Escherichia coli* β-galactosidase, green coffee bean α-galactosidase, *Helix pomatia* β-mannosidase, Jack bean α-mannosidase and β-hexosaminidases from human placenta, bovine kidney and Jack bean. Only the hexosaminidase activity was significantly affected at concentrations below 1 mM of the sp^2^-iminosugars (Supplementary Table 1). The corresponding Lineweaver-Burk plots were consistent with a competitive inhibition mode in all cases. In the thiourea series, the inhibition constants (*K*_i_) values for the human placenta enzyme were in the low micromolar range (3.0 to 16.0 µM) for the *N’*-alky substituted members **1**–**3** and down to nM range (0.24 µM) for the *N’*-phenyl adduct **4**. The notably higher inhibition potency of the aromatic thiourea correlates with its higher hydrogen bond-donating character, strongly suggesting the direct implication of the N’H proton in enzyme-inhibitor complex stabilisation.

The influence of the substituent’s nature in the inhibition properties of the bicyclic iminothiazolidine-type sp^2^-iminosugars (**5**–**8**) was much less pronounced as compared with the monocyclic thiourea counterparts (**1**–**4**). The basic character of the isothiourea group (p*K*_a_ 8–9) implies that it will be fully protonated at neutral pH in all cases^71^. Electrostatic interactions are then expected to be dominant at this region of the molecule. Indeed, the available structural information on β-*N*-acetylhexosaminidase reveals the presence of multiple negative charges at the vicinity of the enzyme active site^29^^,^^32^^,^^72–74^, which is consistent with the much lower *K*_i_ values for the isothioureas as compared with the corresponding neutral thioureas, with the notable exception of the *N’*-phenylthiocarbamoyl DGJNAc derivative **4** for the plant hexosaminidase (*K*_i_ 0.065 µM; Supplementary Table 1).

### 2.3. Inhibition properties against human lysosomal hex in fibroblast lysates

The β-hexosaminidase from human placenta has been previously used in high throughput screenings to identify inhibitors that could act as pharmacological chaperones against disease-associated hexosaminidase mutants^27^. However, it was recently encountered that the inhibitory activity of glycomimetic derivatives towards lysosomal β-hexosaminidases from different tissues can by strikingly different, probably because of variances in the proportions of the mature and precursor forms of the enzyme, which may lead to false positives^62^. Evaluation of the inhibition potency towards total Hex and HexA in human fibroblast lysates represents a more reliable test in search for chaperone candidates against TSD. The corresponding IC_50_ or *K*_i_ values values for **1**–**8** are collected in Table 1. The inhibitory strength followed a similar trend to that encountered for the human placenta enzyme, that is, the bicyclic basic isothioureas **5**–**8** exhibited much higher inhibition efficiencies than the corresponding neutral thioureas **1**–**4**. Among the later, only the *N’*-phenyl derivative **4** showed an IC_50_ value in the low micromolar range against total Hex (10.9 µM). The inhibitory potency generally increased when considering HexA, the target glycosidase in TSD, which was particularly noticeable for the neutral thiourea derivatives. Thus, the *N’*-butyl and *N’*-phenylthioureas **1** and **4** behaved as potent inhibitors of HexA (*K*_i_ 4.7 and 0.91 µM, respectively, at pH 7), in addition to the bicyclic compounds **5**–**8** (0.41 to 0.99 µM at pH 7).

**Table 1.** IC_50_ or *K*_i_ values for sp^2^-iminosugars **1**–**8** human lysosomal hexosaminidases and recombinant human O-GlcNAcase.^a^

	Human enzymes		
	IC_50_ (µM)	*K*_i_ (µM)	
Compound	Total Hex^b^	HexA^b^ pH 7/pH 5	OGA
*DGJNAc thioureas*			
** 1**	>100	4.7/33.7	>100
** 2**	>100	>100/>100	>100
** 3**	>100	>100/>100	>100
** 4**	10.9	0.91/9.4	>100
*DGJNAc isothioureas*			
** 5**	2.6	0.99/9.8	>100
** 6**	1.3	0.58/6.4	>100
** 7**	2.2	0.66/6.9	25
** 8**	0.33	0.41/3.9	>100

^a^IC_50_ and *K*_i_ data are the mean of three independent determinations; errors are in the range of 5–10% and are omitted for clarity. Inhibition was competitive in all cases. No inhibition was observed for any compound at 200 μM on lysosomal α-glucosidase, α- and β-galactosidases and α- and β-mannosidases in cell lysates.

^b^Determined in human fibroblast lysates.

It is worth remarking that the rescuing capabilities of a PC depend not only on the binding potency to the target misfolded enzyme but also on the dissociation rate of the PC-enzyme complex once trafficking to the lysosome has been restored, so that substrate processing can take over. Interestingly, all the tested compounds exhibited HexA inhibition potencies that were about one order of magnitude higher at neutral (mimicking the endoplasmic reticulum) as compared to acidic pH (mimicking the lysosome environment), which is generally considered a good indication for PC candidates (Table 1)^75^. Regarding the HexA/OGA selectivity ratio, except for the *N*’-benzyliminothiazolidine derivative **7** (IC_50_ 0.66 vs 25 µM), it was over 1000-fold for all compounds (IC_50_ against OGA >100 µM).

**Figure 3.**
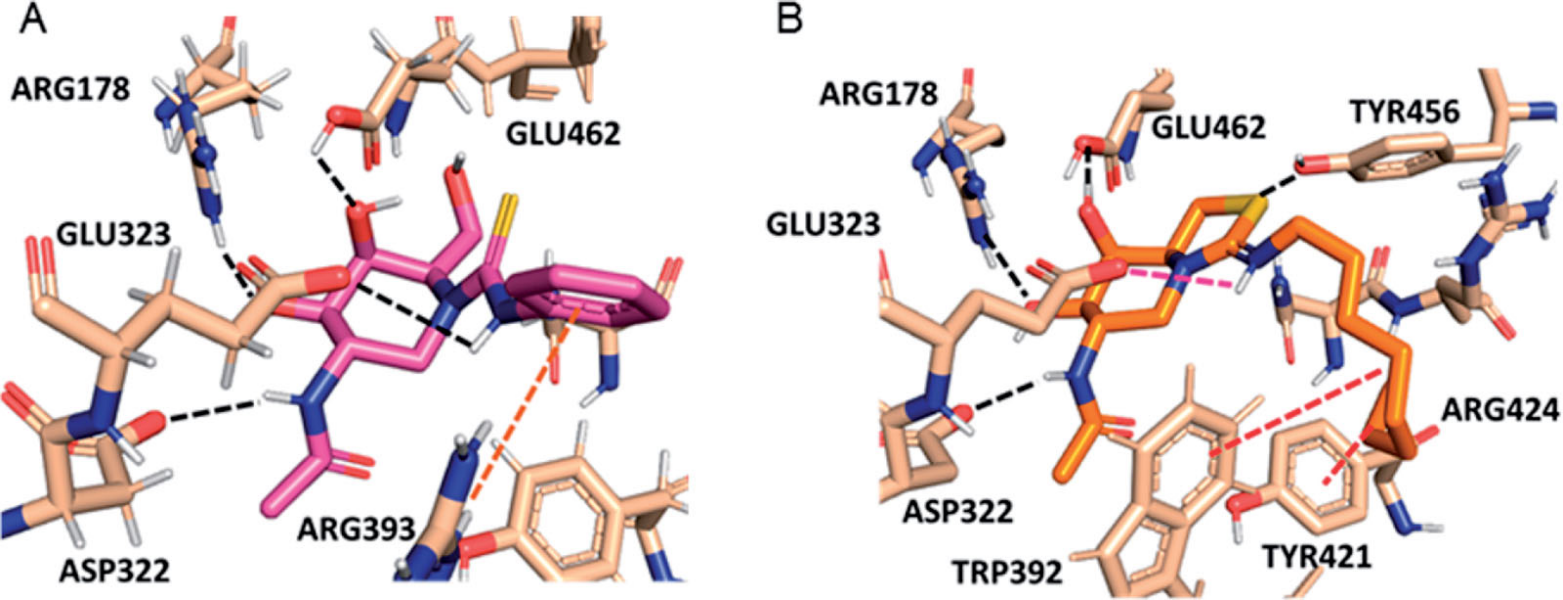
Predicted binding modes of phenylthiourea **4** (A; magenta sticks) and protonated octyliminothiazolidine **6** (B; orange sticks) to HexA (coordinates from PDB ID 2GK1; tan sticks) from the molecular docking experiments. Hydrogen atoms from ligands are not shown for clarity. Hydrogen bonds are represented as black dashed lines, and hydrophobic interactions as red dashed lines.

The superior HexA inhibitory capabilities of the aromatic thiourea **4** and the thiazolidine derivatives **5**–**8** strongly suggest the involvement of the N’H proton of the former (a better H-bond donor as compared to aliphatic thiourea protons^76^^,^^77^) and of the protonated imonium group of the later in hydrogen bonding/salt bridge interactions stabilising the HexA-inhibitor complex. Similar scenarios have been previously reported for other sp^2^-iminosugar/glycosidase pairs^58^^,^^78–81^. Actually, docking experiments support that compound **4** bind to the active site of HexA in a similar manner as NAG-thiazoline in the reported crystal structure.^73^ Specifically, the H-bond network involving the NHAc and the secondary hydroxyl groups with ASP322, ARG178 and GLU462 is conserved. The C(=S)―N’HPh bond adopts the *Z*-configuration, with the N’H proton close to the carboxylate group of the catalytic residue GLU323, forming an angle compatible with the presence of an H-bond (Figure 3A). A parallel situation is encountered for compound **6**, but now the *Z*-oriented octyl chain must twist to avoid steric clashes and occupy a hydrophobic pocket flanked by TRP392 and TYR421 (Figure 3B). Such twisted conformation becomes less favourable in the aliphatic thiourea series, resulting in a destabilisation of the complex that intensifies with the volume of the N’H substituent. Altogether, the data are in agreement with the observed experimental inhibition potency trend: **4**–**8 **>** 1 **≫ **2** and **3**.

### 2.4. Chaperoning vs inhibitory properties in normal and late onset TSD fibroblasts

**Figure 4.**
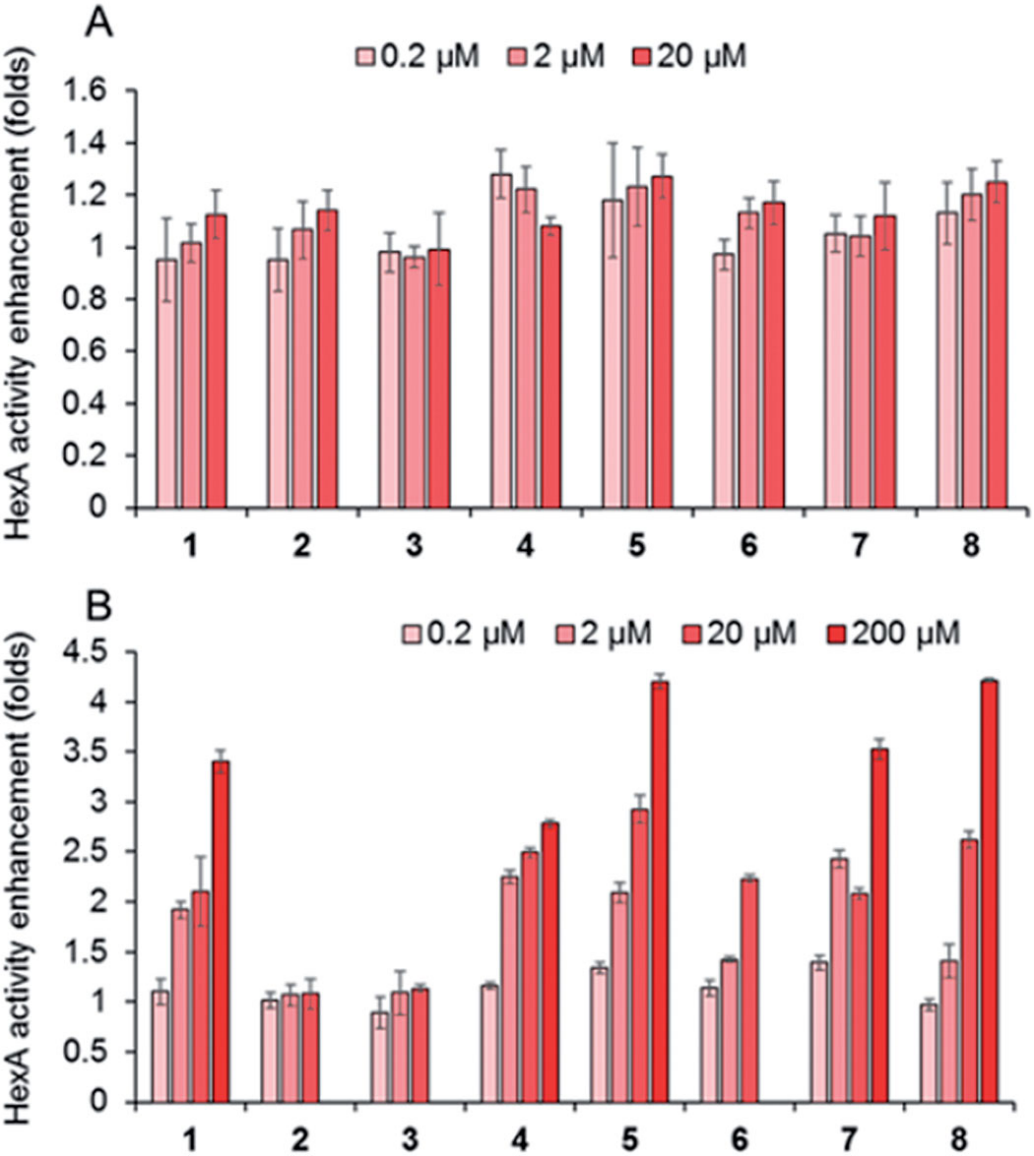
Effect in the HexA activity of different concentrations of the thioureas DGJNAc thioureas **1**–**4** and the isothiourea derivatives **5**–**8** in wild type (A) and G269S TSD fibroblasts (B) relative to controls in the absence of any compound. Bars represent average values ± SD (*n* = 3).

The new PC candidates were further evaluated about the HexA activity effect in wild type (healthy) fibroblasts (Figure 4A) and in TSD fibroblasts from patients hosting the G269S mutation (TSD-GS fibroblasts), the most commonly identified allele in patients with adult-onset TSD and a privileged target for future treatments (Figure 4B).^70^ None of the compounds affected significantly normal HexA activity in the healthy cells. Sharply differently, the results showed that the DGJNAc thioureas **1** and **4** and isothioureas **5**–**8** behaved as very efficient mutant HexA effectors, being able to elicit activity enhancements in the 200%–300% range at 20 µM in all cases and in the 200%–250% range at only 2 µM concentration in the case of compounds **1**, **4**, **5**, **7** and **8**. The later compounds were further evaluated at 200 µM to ascertain whether the inhibitory character compensates the rescuing abilities at high dose. We keep in mind that reaching useful PC concentrations in the central nervous system generally entails much higher doses in plasma and peripheral organs. The data evidenced a further enhancement of the G269S mutant HexA activity in all cases, reaching 3.4- and 2.7-fold for the thioureas **1** and **4** and up to 4.2-, 3.5- and 4.3-fold for the isothioureas **5**, **7** and **8**, respectively. The fact that the effector properties of the compounds prevails in a broad range of concentrations supports the notion that they can diffuse out of the lysosome and avoid intralysosomal accumulation, presumably due to their amphiphilic nature. The results highlight the promise of DGJNAc sp^2^-iminosugars as PCs for late-onset TSD.

**Figure 5.**
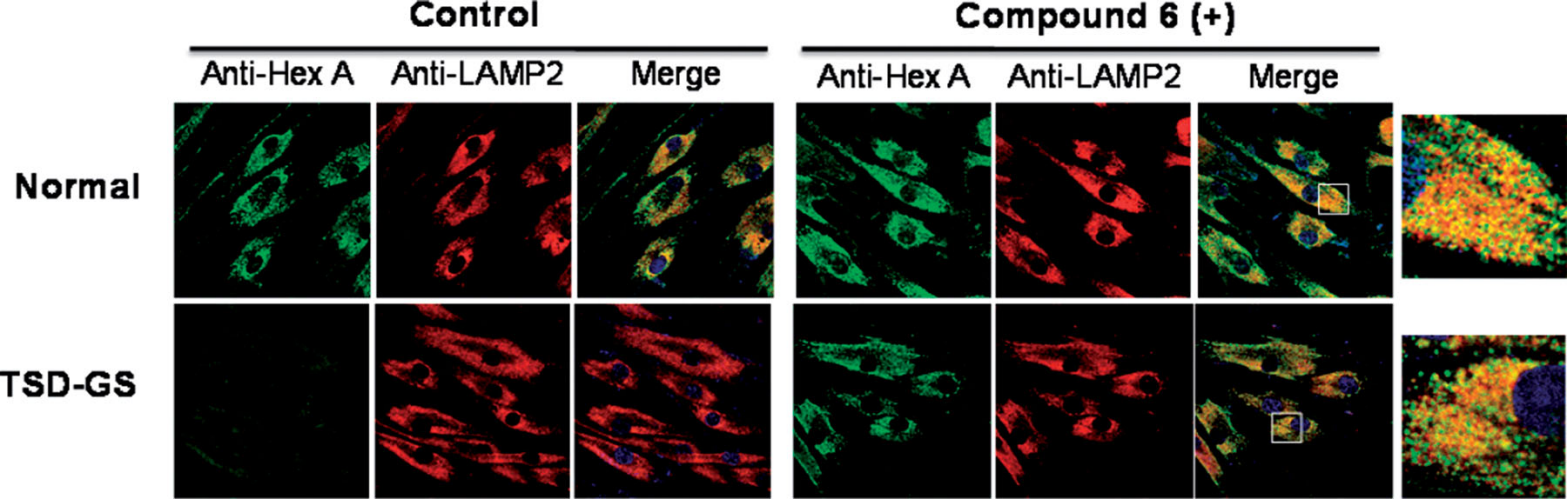
Effect of compound **6** on the traffic of HexA to lysosomes in WT and in TSD-GS fibroblast. Fibroblasts were treated with 20 µM complexed chaperone. Lysosomal marker (lysosome associated membrane protein 2, LAMP-2). HexA is visualised in green; in the merged images, yellow denotes colocalization in lysosomes. The shown pictures are representative of more than 100 cells in 10 randomly obtained images.

To confirm that the mechanism of action involves rescuing and trafficking restoration of the endogenous mutant HexA, immunolabeling and colocalization experiments were further conducted using fibroblast from TSD-GS patients bearing the p.G269S/c.1278insTACT mutation (Figure 5). The left panel shows a series of confocal fluorescence microscopy images after immunostaining of HexA (green), lysosomes (lysosome associated membrane protein 2, red) and the corresponding merge images, in healthy control fibroblast and in TSD-GS fibroblasts. The right panel are the corresponding panels after treatment with **6** at 20 µM. It can be observed that the HexA content in the cells of TD patients is much lower than in the control and that it hardly colocalizes with the lysosomes. After treatment with chaperone **6**, the amount of HexA increased significantly and clearly colocalized with this organelle as it happens in the control cells (Figure 5, Supplementary Figure 1). These results complement the *in vitro* assays and strongly supports that the observed enzyme activity enhancements correspond undoubtedly to the lysosomal HexA. Given that the residual activity of G269S mutant HexA was about 6% that of wild-type HexA,^26^ the measured enhancements warrant substrate processing levels well over the 10% of the normal threshold, considered critical for a medically relevant effect, in the 2–200 µM range, with no apparent toxicity. By comparison, pyrimethamine afforded 1.2 and 2.0-fold G269S mutant HexA activity enhancements at 0.5 and 1.0 g/mL in parallel assays, higher concentrations resulting in significant toxicity.

## 3. Conclusion

Grounding on the current structural knowledge on hexosaminidase-substrate (or inihibitor) complexes, we hypothesised that sp^2^-iminosugars emulating the substitution profile and stereochemistry of GalNAc would fit in the active site of HexA but not in that of the related enzyme OGA. By equipping the glycomimetic core with suitable appendages, providing additional contacts with the enzyme, the ability to properly folding and rescuing mutant HexA causative of late-onset TSD could be optimised. To put this concept into practice, we conducted the direct conjugation of four different isothiocyanates with DGJNAc. The corresponding thiourea adducts were thus obtained and subsequently subjected to intramolecular cyclisation to afford bicyclic pyridine-thiazolidine derivatives. Nanomolar competitive inhibitors of HexA at pH 7, with high HexA/OGA selectivity, were identified in both the (neutral) thiourea and (basic) iminothiazolidine series. The inhibitory potency fell down by ten-fold at pH 5, which should facilitate the dissociation of the HexA-glycomimetic complex at the lysosome, where the Hex substrate (namely G_M2_ ganglioside) accumulates. In agreement with this notion, remarkable HexA activity enhancements were achieved in fibroblasts from TSD patients having the G269S mutation, associated to the late-onset phenotype. Interestingly, most candidates still enhanced G269S HexA activity in cell assays even at high (200 µM) concentration. Altogether, the results validate the approach of using GalNAc sp^2^-iminosugar mimics to identify drug-like pharmacological chaperone candidates for the treatment of late-onset TSD.

## 4. Experimental section

### 4.1. General methods

Reagents and solvents were purchased from commercial sources and used without further purification. Optical rotations were measured with a JASCO P-2000 polarimeter, using a sodium lamp (λ = 589 nm) at 22 °C in 1 cm or 1 dm tubes. IR spectra were recorded on a JASCO FTIR-410 device. UV spectra were recorded on JASCO V-630 instrument; unit for ε values: mM^−1 ^cm^−1^. NMR experiments were performed at 300 (75.5) and 500 (125.7) MHz. 1-D TOCSY as well as 2-D COSY and HMQC experiments were carried out to assist signal assignment. In the FABMS spectra, the primary beam consisted of Xe atoms with a maximum energy of 8 keV. The samples were dissolved in *m*-nitrobenzyl alcohol or thioglycerol as the matrices and the positive ions were separated and accelerated above a potential of 7 keV. NaI was added as cationizing agent. For ESI mass spectra, 0.1 pm sample concentrations were used, the mobile phase consisting of 50% aq MeCN at 0.1 ml min^−1^. Thin-layer chromatography was performed on precoated TLC plates, silica gel 30 F-245, with visualisation by UV light and also with 10% H_2_SO_4_ or 0.2% w/v cerium (IV) suphate-5% ammonium molybdate in 2 m H_2_SO_4_ or 0.1% ninhydrin in EtOH. Column chromatography was performed on Chromagel (silice 60 AC.C 70–200 µm). All compounds were purified to ≥95% purity as determined by elemental microanalysis results obtained on a CHNS-TruSpect® Micro elemental analyser (Instituto de Investigaciones Químicas de Sevilla, Spain) from vacuum-dried samples. The analytical results for C, H, N and S were within ±0.5 of the theoretical values. Starting 2-acetamido-1,2-dideoxygalactonojirimycin (DGJNAc) was prepared following a described procedure^64^. 4-Methylumbeliferone (4-MU)-conjugated *N*-acetyl-β-D-glucosaminide was obtained from Sigma (St. Louis, MO). 4-MU-*N*-acetyl-β-D-glucosaminide-6-sulfate sodium salt was from Slater and Frith Ltd (Norfolk, UK). LDH cytotoxicity kit was from WAKO (Tokyo, Japan).

### 4.2. Chemistry

#### 4.2.1. General procedure for the preparation of 2-acetamido-N-(N’-alkyl(aryl)thiocarbamoyl)-1,2-dideoxygalactonojirimycin derivatives (1–4)

To a solution of DGJNAc (102 mg, 0.423 mmol) in pyridine (3.8 ml), Et_3_N (0.117 ml, 0.846 mmol) and the corresponding isothiocyanate (butyl, octyl, benzyl or phenyl isothiocyanate; 0.508 mmol) were added. The mixture was stirred at rt for 7–20 h. The solvent was coevapored with toluene and the resulting residue was purified by column chromatography using the eluent indicated in each case to afford the target DGJNAc thiourea derivatives.

2-Acetamido-1,2-dideoxy-5-*N*-(*N’*-butylthiocarbamoyl)galactonojirimycin **(1**). Column chromatography, eluent 100:10:1 DCM-MeOH-H_2_O. Yield: 106.7 mg (79%). [α]_D_ +6.7 (*c* 0.38, H_2_O). R*_f_* 0.41 (70:10:1 DCM-MeOH-H_2_O). UV (H_2_O) 242 nm (ε_mM_ 12.3). ^1^H NMR (300 MHz, CD_3_OD): δ 4.77 (bd, 1 H, *J*_1a,1b_=14.3 Hz, H-1a), 4.42 (m, 1 H, H-5), 4.03 (dd, 1 H, *J*_6a,6b_=11.7 Hz, *J*_5,6a_=8.8 Hz, H-6a), 3.95 (m, 3 H, H-2, H-4, H-6b, 3.76 (dd, 1 H, *J*_2,3_=4.4 Hz, *J*_3,4_=2.8 Hz, H-3), 3.57 (m, 2 H, C*H_2_*NH, H-1b), 1.96 (s, 1 H, COCH_3_), 1.59 (m, 2 H, CH_2_), 1.39 (m, 2 H, CH_2_), 0.95 (t, 3 H, *J*_H,H_=7.3 Hz, CH_3_). ^13 ^C NMR (75.5 MHz, CD_3_OD): δ 186.9 (CS), 173.3 (CO), 71.7 (C-3), 68.4 (C-4), 64.3 (C-5), 60.3 (C-6), 53.5 (C-2), 46.7(CH_2_N), 44.2 (C-1), 32.2 (CH_2_), 22.8 (CO*C*H_3_), 21.1 (CH_2_), 14.1 (CH_3_). ESIMS: *m/z* 342.2 ([M + Na]^+^). Anal. Calcd for C_13_H_25_N_3_O_4_S: C, 48.88; H, 7.89; N, 13.16; S, 10.04. Found: C, 48.96; H, 7.94; N, 12.91; S 9.72.

2-Acetamido-1,2-dideoxy-5-*N*-(*N’*-octylthiocarbamoyl)galactonojirimycin **(2**). Column chromatography, eluent 80:10:1 DCM-MeOH-H_2_O. Yield: 135 mg (85%). [α]_D_ −149.4 (*c* 0.80, MeOH). R*_f_* 0.55 (70:10:1 DCM-MeOH-H_2_O). UV (MeOH) 249 nm (ε_mM_ 11.5). ^1^H NMR (500 MHz, CD_3_OD): δ 4.81 (bd, 1 H, *J*_1a,1b_=15.4 Hz, H-1a), 4.47 (m, 1 H, H-5), 4.06 (dd, 1 H, *J*_6a,6b_=11.8 Hz, *J*_5,6a_=9.1 Hz, H-6a), 4.01 (m, 1 H, H-2), 3.97 (m, 2 H, H-4, H-6b), 3.79 (dd, 1 H, *J*_2,3_=4.2 Hz, *J*_3,4_=2.9 Hz, H-3), 3.58 (m, 2 H, C*H_2_*NH, H-1b), 1.99 (s, 1 H, COCH_3_), 1.63 (m, 2 H, CH_2_), 1.37 (m, 10 H, CH_2_), 0.93 (t, 3 H, *J*_H,H_=7.2 Hz, CH_3_). ^13 ^C NMR (125.7 MHz, CD_3_OD): δ 186.7 (CS), 173.3 (CO), 71.6 (C-3), 68.3 (C-4), 64.2 (C-5), 60.2 (C-6), 53.4 (C-2), 47.0 (CH_2_N), 44.1 (C-1), 33.0, 30.4, 30.3, 30.1, 28.0, 23.7 (CH_2_), 22.7 (CO*C*H_3_), 14.4 (CH_3_). FABMS: *m/z* 398 (100, [M + Na]^+^). Anal. Calcd for C_17_H_33_N_3_O_4_S: C, 54.37; H, 8.86; N, 11.19; S, 8.54. Found: C, 54.28; H, 8.76; N, 10.89; S 8.22.

2-Acetamido-1,2-dideoxy-5-*N*-(*N’*-benzylthiocarbamoyl)galactonojirimycin **(3**). Column chromatography, eluent 100:10:1 DCM-MeOH-H_2_O. Yield: 96 mg (80%). [α]_D_ ­165.9 (*c* 0.88, MeOH). R*_f_* 0.42 (70:10:1 DCM-MeOH-H_2_O). UV (MeOH) 210 nm (ε_mM_ 22.6). ^1^H NMR (300 MHz, CD_3_OD): δ 7.35–7.21 (Ph), 4.89 (bd, 1 H, *J*_1a,1b_=15.0 Hz, H-1a), 4.79 (m, 2H, CH_2_Ph), 4.57 (m, 1H, H-5), 4.06 (dd, 1 H, *J*_6a,6b_=11.8 Hz, *J*_5,6a_=9.1 Hz, H-6a), 3.95 (m, 3 H, H-2, H-4, H-6b), 3.77 (dd, 1 H, *J*_2,3_=4.0 Hz, *J*_3,4_=2.9 Hz, H-3), 3.65 (dd, 1 H, H-1b), 1.96 (s, 1 H, COCH_3_). ^13 ^C NMR (125.7 MHz, CD_3_OD): δ 187.1 (CS), 173.3 (CO), 139.9, 129.5, 128.7, 128.2 (Ph), 71.5 (C-3), 68.2 (C-4), 64.1 (C-5), 60.1 (C-6), 53.3 (C-2), 50.6 (*C*H_2_Ph), 44.3 (C-1), 22.8 (CO*C*H_3_). ESIMS: *m/z* 373.3 ([M + Na]^+^). Anal. Calcd for C_16_H_23_N_3_O_4_S: C, 54.37; H, 6.56; N, 11.89; S, 9.07. Found: C, 54.13; H, 6.28; N, 11.90; S 9.22.

2-Acetamido-1,2-dideoxy-5-*N*-(*N’*-phenylthiocarbamoyl)galactonojirimycin **(4**). Column chromatography, eluent 100:10:1 DCM-MeOH-H_2_O. Yield: 98.7 mg (69%). [α]_D_ −235.9 (*c* 0.92, MeOH). R*_f_* 0.50 (70:10:1 DCM-MeOH-H_2_O). UV (MeOH) 260 nm (ε_mM_ 32.8). ^1^H NMR (300 MHz, CD_3_OD): δ 7.42–7.09 (m, 5H, Ph), 4.9 (bd, 1 H, *J*_1a,1b_=15.6 Hz, H-1a), 4.66 (m, 1 H, H-5), 4.15 (dd, 1 H, *J*_6a,6b_=11.7 Hz, *J*_5,6a_=9.3 Hz, H-6a), 4.04 (m, 3 H, H-2, H-4, H-6b), 3.80 (dd, 1 H, *J*_2,3_=4.5 Hz, *J*_3,4_=2.7 Hz, H-3), 3.69 (dd, 1 H, *J*_1b,2_=3.7 Hz, H-1b), 1.99 (s, 1 H, COCH_3_). ^13 ^C NMR (75.5 MHz, CD_3_OD): δ 186.7 (CS), 173.4 (CO), 141.8, 129.4, 125.8, 125.1 (Ph), 71.8 (C-3), 68.5 (C-4), 64.8 (C-5), 60.7 (C-6), 53.4 (C-2), 44.6 (C-1), 22.8 (CO*C*H_3_). ESIMS: *m/z* 361.2 ([M + Na]^+^). Anal. Calcd for C_15_H_21_N_3_O_4_S: C, 53.08; H, 6.24; N, 12.38; S, 9.45. Found: C, 53.22; H, 6.48; N, 12.20; S 9.08.

#### 4.2.2. General procedure for the preparation of 2-acetamido-1,2-dideoxy-5-N,6-S-(N’-alkyl(aryl)iminomethylidene)-6-thiogalactonojirimycin derivatives (5–8)

The DGJNAc thiourea derivative (**1–4**, 0.256 mmol) was dissolved in MeOH (8.0 ml) and a few drops of concentrated HCl were added until pH 1. The solution was stirred at rt until complete disappearance of the starting material (7–24 h). The solvent was removed under reduced pressure and the residue was coevaporated with MeOH several times until neutral pH and purified by column chromatography using the eluent indicated in each case to afford the corresponding bicyclic DGJNAc isothioureas.

2-Acetamido-1,2-dideoxy-5-*N*,6-*S*-(*N’*-butyliminomethylidene)-6-thiogalactonojirimycin **(5).** Column chromatography, eluent: 70:10:1→ 30:10:1 CH_2_Cl_2_-MeOH-H_2_O. Yield: 78 mg (quantitative). [α]_D_ +44.0 (*c* 0.48, H_2_O). R*_f_* 0.40 (40:10:1 CH_2_Cl_2_-MeOH-H_2_O). ^1^H NMR (500 MHz, CD_3_OD): δ 4.45 (td, 1 H, *J*_5,6_=8.4 Hz, *J*_4,5_=1.3 Hz, H-5), 4.15 (m, 2 H, H-1a, H-2), 3.98 (m, 1 H, H-4), 3.74 (dd, 1 H, *J*_2,3_=10.3 Hz, *J*_3,4_=2.6 Hz, H-3), 3.66 (m, 2 H, H-6), 3.36 (m, 2 H, C*H_2_*N), 3.00 (dd, 1 H, *J*_1a,1b_=12.5 Hz, *J*_1b,2_=11.0, H-1b), 2.01 (s, 1 H, COCH_3_), 1.67 (m, 2 H, CH_2_), 1.41 (m, 2 H, CH_2_), 0.97 (t, 3 H, *J*_H,H_=7.4 Hz, CH_3_). ^13 ^C NMR (75.5 MHz, CD_3_OD): δ 174.1 (CO), 172.5 (CN), 72.7 (C-3), 70.9 (C-4), 68.9 (C-5), 49.6 (CH_2_N), 47.1 (C-2), 46.7 (C-1), 32.1 (CH_2_), 29.3 (C-6), 22.8 (CO*C*H_3_), 20.7 (CH_2_), 13.9 (CH_3_). ESIMS: *m/z* 302.2 [M + H]^+^. Anal. Calcd for C_13_H_23_N_3_O_3_S: C, 51.80; H, 7.69; N, 13.94; S, 10.64. Found: C, 51.99; H, 7.64; N, 13.77; S, 10.36.

2-Acetamido-1,2-dideoxy-5-*N*,6-*S*-(*N’*-octyliminomethylidene)-6-thiogalactonojirimycin **(6).** Column chromatography, eluent: 40:10:1 DCM-MeOH-H_2_O. Yield: 82.7 mg (96%). [α]_D_ +34.4 (*c* 1.05, MeOH). R*_f_* 0.29 (40:10:1 DCM-MeOH-H_2_O). ^1^H NMR (500 MHz, CD_3_OD): δ 4.42 (td, 1 H, *J*_5,6_=7.1 Hz, *J*_4,5_=1.4 Hz, H-5), 4.14 (m, 2 H, H-1a, H-2), 3.96 (m, 1 H, H-4), 3.71 (dd, 1 H, *J*_2,3_=10.2 Hz, *J*_3,4_=2.6 Hz, H-3), 3.64 (m, 2 H, H-6), 3.35 (t, 2 H, C*H_2_*N), 2.96 (dd, 1 H, *J*_1a,1b_=12.5 Hz, *J*_1b,2_=10.9 Hz, H-1b), 2.01 (s, 1 H, COCH_3_), 1.68 (m, 2 H, CH_2_), 1.33 (m, 10 H, CH_2_), 0.91 (t, 3 H, *J*_H,H_=7.0 Hz, CH_3_). ^13 ^C NMR (125.7 MHz, CD_3_OD): δ 174.1 (CO), 172.4 (CN), 72.7 (C-3), 71.0 (C-4), 68.9 (C-5), 50.0 (CH_2_N), 47.1 (C-2), 46.7 (C-1), 29.2 (C-6), 32.9, 30.2, 30.2, 30.0, 27.6, 23.7 (CH_2_), 22.8 (CO*C*H_3_), 14.4 (CH_3_). ESIMS: *m/z* 358 [M + H]^+^. Anal. Calcd for C_17_H_31_N_3_O_3_S: C, 57.11; H, 8.74; N, 11.75; S, 8.97. Found: C, 57.28; H, 8.66; N, 11.47; S, 9.14.

2-Acetamido-1,2-dideoxy-5-*N*,6-*S*-(*N’*-benzyliminomethylidene)-6-thiogalactonojirimycin **(7).** Column chromatography, eluent: 60:10:1→ 40:10:1 DCM-MeOH-H_2_O. Yield: 60 mg (quantitative). [α]_D_ +48.8 (*c* 1.0, MeOH). R*_f_* 0.25 (40:10:1 DCM-MeOH-H_2_O). ^1^H NMR (500 MHz, CD_3_OD): δ 7.38–7.29 (Ph), 4.52 (s, 2 H, C*H*_2_Ph), 4.27 (bt, 1 H, *J*_1a,1b_=8.4 Hz, H-1a), 4.17 (m, 2 H, H-2, H-5), 3.95 (m, 1 H, H-4), 3.59 (dd, 1 H, *J*_6a,6b_=11.0 Hz, *J*_5,6a_=8.2 Hz, H-6a), 3.55 (dd, 1 H, *J*_5,6b_=8.7 Hz, H-6b), 2.88 (td, 1H, *J*_1b,2_=3.4 Hz, H-1b), 1.99 (s, 3H, COCH_3_). ^13 ^C NMR (125.7 MHz, CD_3_OD): δ 174.1 (CO), 171.2 (CN), 137.5, 129.8, 129.1, 128.7 (Ph), 73.1 (C-3), 70.9 (C-4), 68.2 (C-5), 53.9 (*C*H_2_Ph), 47.3 (C-2), 47.0 (C-1), 29.2 (C-6), 22.7 (CO*C*H_3_). ESIMS: *m/z* 358.3 ([M + Na]^+^), 336.3 ([M + H]^+^). Anal. Calcd for C_16_H_21_N_3_O_3_S: C, 57.29; H, 6.31; N, 12.53; S, 9.56. Found: C, 57.01; H, 6.12; N, 12.20; S, 9.29.

2-Acetamido-1,2-dideoxy-5-*N*,6-*S*-(*N’*-phenyliminomethylidene)-6-thiogalactonojirimycin **(8).** Column chromatography, eluent: 80:10:1→ 40:10:1 DCM-MeOH-H_2_O. Yield: 27 mg (41%). [α]_D_ +100.2 (*c* 0.28, MeOH). R*_f_* 0.63 (40:10:1 DCM-MeOH-H_2_O). ^1^H NMR (300 MHz, 10:1 CD_3_CN:D_2_O): δ 7.29–6.87 (Ph), 4.15 (dd, 1 H, *J*_1a,1b_=12.6 Hz, *J*_1a,2_=5.4 Hz, H-1a), 4.03 (m, 1 H, H-2), 3.88 (m, 1H, H-4), 3.73 (m, 1 H, H-5), 3.58 (dd, 1 H, *J*_2,3_=10.6, *J*_3,4_=2.9 Hz, H-3), 3.29 (dd, 1H, *J*_6a,6b_=10.8 Hz, *J*_5,6a_=9.3 Hz, H-6a), 3.14 (dd, 1 H, *J*_5,6b_=7.6 Hz, H-6b), 2.56 (dd, 1H, *J*_1b,2_=11.1 Hz, H-1b), 1.93 (s, 3H, COCH_3_). ^13 ^C NMR (75.5 MHz, 10:1 CD_3_CN:D_2_O): δ 174.2 (CO), 162.8 (CN), 129.9, 124.6, 123.3 (Ph), 73.7 (C-3), 69.8 (C-4), 64.0 (C-5), 47.2 (C-2), 46.6 (C-1), 27.9 (C-6), 23.1 (CO*C*H_3_). ESIMS: *m/z* 344.3 ([M + Na]^+^), 322.3 ([M + H]^+^). Anal. Calcd for C_15_H_19_N_3_O_3_S: C, 56.06; H, 5.96; N, 13.07; S, 9.98. Found: C, 55.73; H, 6.03; N, 12.82; S, 9.85.

### 4.3. Molecular docking

The protein structure of HexA was obtained from Protein Data Bank (PDB code: 2GK1, heterodimer consisting of α and β subunits complexed with two NAG-thiazoline molecules). The protein-ligand docking calculations were performed with the AutoDock Vina software^82^. The protein was prepared following the Protein Preparation Wizard of Schrödinger Software in Maestro^83^. Hydrogen atoms were added under physiological conditions (pH 7). The initial coordinates of the ligands **4** and protonated **6** were built with the Maestro program, assuming a ^4^*C*_1_ conformation for the six-membered ring. A previous energy minimisation using OPLS3e force field was used to optimise the ligand structures. The docking calculations and the volume for exploration was strategically defined by a grid box centred at the NAG-thiazoline binding site (x= −9.659, y = 52.904, z = 28.392, according to PDB ID 2GK1 coordinates), with 54 × 54 × 54 points (20 × 20 × 20 Å) and a grid spacing of 0.375 Å. Kollman charges were added to the protein using AutodockTools 1.5.6 software. The docking calculations were carried out using the Lamarckian genetic algorithm, with rigid side chains for the receptor and randomly changing the torsion angles (8 rotable bonds for compound **4** and 13 rotable bonds for compound **6**) and overall orientation of the ligand. Autodock Vina generated docking results for the top 9 poses in the pdbqt format for each ligand and the data were analysed in Pymol. For comparison, docking calculations of protonated NAG-thiazoline that reproduced both the binding conformation and hydrogen bonds of NAG-thiazoline in a cocrystallized structure (pdb: 2GK1) was carried out (Supplementary Figure 10). Molecular representations in figures were generated with the Pymol software.

### 4.4. Commercial enzyme inhibition assays

Inhibition constant (*K*_i_) values were determined by spectrophotometrically measuring the residual hydrolytic activities of the glycosidases against the respective *o*- (for β-galactosidase from *E. coli*) or *p*-nitrophenyl α- or β-D-glycopyranoside (for other glycosidases) in the presence of the iminosugars. Each assay was performed in phosphate buffer or phosphate-citrate buffer (for α- or β-mannosidase, amyloglucosidase or β-*N*-acetylglucosaminidase) at the optimal pH for the enzymes. The reactions were initiated by addition of the enzyme to a solution of the substrate in the absence or presence of various inhibitor concentrations. The mixture was incubated for 10–30 min at 37 °C or 55 °C (for amyloglucosidase) and the reaction was quenched by addition of 1 m Na_2_CO_3_. Reaction times were appropriate to obtain 10%–20% conversion of the substrate in order to achieve linear rates. The absorbance of the resulting mixture was determined at 405 nm. Approximate values of *K*_i_ were determined using a fixed concentration of substrate (around the *K*_M_ value for the different glycosidases) and various inhibitor concentrations. Full *K*_i_ determinations and the enzyme inhibition mode were determined from the slope of Lineweaver-Burk plots and double reciprocal analysis.

### 4.5. Profiling of the inhibitory selectivity towards lysosomal glycosidases by in vitro enzyme assay using human fibroblast lysates

For determination of lysosomal enzyme activities in cell lysates, cells were scraped into ice-cold H_2_O (10^6^ cells mL^−1^) and lysed by sonication. Insoluble materials were removed by centrifugation at 15,000 rpm for 5 min and protein concentrations were determined with Protein Assay Rapid Kit (WAKO, Tokyo, Japan). 10 µL of the lysates in 0.1% Triton X-100 in distilled water were incubated at 37 °C with 20 µL of the substrate solution in 0.1 M citrate buffer, pH 4.5, in absence or presence of increasing concentrations of the chaperones. The substrates were 4-methylumbelliferone (4-MU)-conjugated β-D-glucopyranoside (for GCase), α-D-glucopyranoside (for α-glucosidase), α-D-galactopyranoside (for α-Galase), β-D-galactopyranoside (for β-galactosidase), *N*-acetyl-β-D-glucosaminide (for total β-hexosaminidase), *N*-acetyl-β-D-glucosaminide-6-sulfate sodum salt (for HexA) and α-*N*-acetyl-D-galactosaminide for α-*N*-acetylgalactosaminidase. HexA activities in cell lysates were also determined in 0.1 M citrate buffer at pH 5 or pH 7, supplemented with sodium taurocholate (0.8% w/v). The reactions were terminated by adding 0.2 ml of 0.2 M glycine sodium hydroxide buffer (pH 10.7). The liberated 4-methylumbelliferone was measured in the black-well plate with a Perkin Elmer Luminescence Spectrometer (excitation wavelength: 340 nm; emission: 460 nm). One unit of enzyme activity was defined as nmol of 4-methylumbelliferone released per hour and normalised for the amount of protein contained in the lysates.

### 4.6. Cloning, expression and purification of hOGA and inhibition assay

The gene of the longest isoform of hOGA (hOGA-L; Uniprot Accession number: O60502) was synthesised in a codon-optimised form for recombinant expression in *Escherichia coli*. The successful expression construct encoded the N-terminal region of hOGA, comprising amino acids 11–396, and the C-terminal region, comprising amino acids 535–715. The N-terminal construct was cloned in the vector pACYC-Duet (Millipore) using the sequence and ligation-independent cloning method in frame with an N-terminal His6 tag. The C-terminal construct was cloned into the vector pET-YSBLIC3C27 with an N-terminal His6 tag followed by a 3 C-protease cleavage site using the same method. The nucleotide sequences of all made constructs were confirmed by sequencing. Both vectors were simultaneously transformed into *E. coli* Bl21(DE3)-Gold (Agilent) for subsequent protein expression. Cells were grown in 2 L TB-medium to an OD600 of ∼1.0 and protein synthesis was then induced by adding IPTG to a final concentration of 0.1 mM. Protein expression was carried out at 16 °C with an induction time of 20 h. The cells were harvested by centrifugation at 4,500 × g for 20 min, flash frozen and stored at −20 °C until required. For purification of the hOGA complex, cells were resuspended in 50 mM HEPES pH 7.0, 750 mM NaCl, 20 mM imidazole, and 0.5 mM DTT (resuspension buffer). Cells were lysed using a French Press at 25 kPsi. The lysate was cleared by centrifugation at 50,000 g for 1 h, and the supernatant was passed through a 10 ml HisTrap FF column (GE Healthcare) pre-equilibrated with resuspension buffer. The bound hOGA was purified by gradient elution over 10 column volumes using 0% to 50% of elution buffer (50 mM HEPES pH 7.0, 750 mM NaCl, 500 mM imidazole and 0.5 mM DTT). hOGA-containing fractions were combined, concentrated by ultrafiltration using Vivaspin columns (Sartorius) with a molecular weight cut-off (MWCO) of 30 kDa, and applied to a Superdex S200 column (GE Healthcare) pre-equilibrated with size-exclusion buffer (10 mM HEPES pH 7.0, 250 mM NaCl, 1 mM DTT). Fractions corresponding to the dimeric form of hOGA were combined and concentrated to 20 mg/mL by ultrafiltration with a Vivaspin (MWCO: 30 kDa) column, flash frozen using liquid nitrogen, and stored at −80 °C until required. Western blotting, conducted as previously reported, showed an intense band corresponding to hOGA and low intensity bands arising from truncated forms of the enzyme (Supplementary Figure 11).

Inhibition potencies for compounds **1**–**8** against hOGA enzyme were determined by measuring the change in fluorescent signal corresponding to the rate of hydrolytic activity against the artificial substrate, resorufin *N*-acetyl-β-D-glucosaminide. hOGA activity assays were performed in a buffer of 20 mM HEPES, 5 mM EDTA, 150 mM KCl, pH 7.1 and 0.2 nM [hOGA]. *K*_m_=25 µM was measured with these conditions. Inhibition assays were run at 0.2 nM [hOGA] in the same buffer, in the presence or absence of various concentrations of inhibitors and at a fixed substrate concentration of 25 µM and 1% DMSO. First, inhibitor was serially diluted to the desired range of concentrations in 2% DMSO buffer. Next, 50 µL of inhibitor solutions at various concentrations in 2% DMSO Buffer was added to 50 μl of 0.8 nM enzyme and allowed to incubate at 25 °C for 5 min. 100 µL of 50 µM and 1% DMSO substrate was then added and reaction mixture was immediately mixed and aliquoted in 45 µL triplicates to a CORNING 384 well black plate. Fluorescence signal was measured continuously for 20 min at 37 °C in a BioTek Neo 2 Plate reader set at excitation and emission wavelengths of 572 and 610 nm, respectively. Max reaction rates for all inhibitor concentrations were calculated within Gen5 BioTek reader software. % Activity was subsequently calculated for each inhibitor concentration against the max reaction rate of uninhibited hOGA. Except for compound **7** (IC_50_ 25 mM), none of the compounds displayed significant hOGA inhibition at concentrations up to 100 µM.

### 4.7. Cell culture and chaperone treatment

Human skin fibroblasts from normal subject were cultured as described. Human fibroblasts from TSD patient with p.G269S/c.1278insTACT *HEXA* mutation were obtained from Coriell Cell Repositories (Camden, NJ). For measurement of chaperone effects, cells were cultured in the medium with or without compounds (**1**–**8**) for 4 days and then the cell lysates were subjected for the lysosomal enzyme assay as described above. The enzyme activities were normalised with protein concentrations measured by protein assay rapid kit (Wako, Tokyo, Japan).

### 4.8. Immunofluorescence microscopy

Immunofluorescence microscopy was performed using standard methods as previously described.40 Coverslips were analysed using a fluorescence microscope (Leica DMRE, Leica Microsystems GmbH, Wetzlar, Germany). Deconvolution studies and three-dimensional projections were performed using a DeltaVision system (Applied Precision, Issaquah, WA) with an Olympus IX-71microscope. The deconvolved images were derived from optical sections taken at 30 nm intervals using a 60× PLAPON objective with a 1.42 numerical aperture. More than 100 cells in 10 randomly obtained images were evaluated in each experiment to confirm reproducibility.

### 4.9. Statistical analysis

All results are expressed as mean ± SD of three independent experiments, each conducted in triplicate. The measurements were statistically analysed using the Student’s *t* test for comparing 2 groups. The level of significance was set at *p* < 0.05.

## Supplementary Material

Supplemental Material
