# Supplementary material for: sp2-Iminosugars targeting human lysosomal β-hexosaminidase as pharmacological chaperone candidates for late-onset Tay-Sachs disease
Source: J Enzyme Inhib Med Chem. 2022 May 16;37(1):1364–74. doi: 10.1080/14756366.2022.2073444 (PMC9126592; doi:10.1080/14756366.2022.2073444)
Supplement: Supplemental Material [file IENZ_A_2073444_SM5352.pdf]

Supporting Information

sp²-Iminosugars Targeting Human Lysosomal β-Hexosaminidase as Pharmacological Chaperone Candidates for Late-Onset Tay-Sachs Disease

Authors:

Manuel González-Cuesta,[a] Irene Herrera-González,[a] M. Isabel García-Moreno,[a] Roger A. Ashmus,[b] David J. Vocadlo,[b,c] José M. García Fernández,[d] Eiji Nanba,[e] Katsumi Higaki,[e,*] Carmen Ortiz Mellet.[a,*]

Affiliations:

[a] Departamento de Química Orgánica, Facultad de Química, Universidad de Sevilla, 41012 Sevilla, Spain; [b] Department of Chemistry, Simon Fraser University, Burnaby, British Columbia, Canada; [c] Department of Molecular Biology and Biochemistry, Simon Fraser University, Burnaby, British Columbia, Canada; [d] Instituto de Investigaciones Químicas (IIQ), CSIC - Universidad de Sevilla, Avda. Américo Vespucio 49, 41092 Sevilla, Spain; [e] Organization for Research Initiative and Promotion, Tottori University, 86 Nishi-cho, Yonago 683-8503, Japan

***Corresponding authors:**

Carmen Ortiz Mellet, mellet@us.es

Katsumi Higaki, kh4060@tottori-u.ac.jp

Table of Contents:

Inhibition assays against commercial β-*N*-acetylhexosaminidases S2

Immunoblot for chaperoning effect of **6** S2

NMR spectra of new DGJNAc derivatives S3-S10

Crystal and docked structure of NAG-thiazoline in complex with HexA S11

Immunoblot of hOGA S11

References S12

Inhibition assays against commercial β-*N*-acetylhexosaminidases (Ki values, µM)

Table S1. K_i or IC_{50} values for sp^2-iminosugars **1-8** against commercial β-*N*-acetylhexosaminidases.

Compound	Commercial β-hexosaminidases (K_i, µM)		
	Human placenta	Bovine kidney	Jack bean
DGJNAc thioureas			
**1**	4.8±0.2	3.5±0.2	3.0±0.1
**2**	3.0±0.1	2.2±0.1	7.2±0.2
**3**	16.0±0.5	6.3±0.2	5.1±0.2
**4**	0.24±0.02	0.14±0.01	0.065±0.003
DGJNAc isothioureas			
**5**	0.055±0.002	0.024±0.002	0.14±0.01
**6**	0.026±0.001	0.015±0.001	0.048±0.002
**7**	0.026±0.001	0.020±0.001	0.088±0.003
**8**	0.044±0.002	0.046±0.002	0.13±0.01

[a] K_i data are presented as the mean ± SD (n = 3). Inhibition was competitive in all cases. No inhibition was observed for any compound at 1 mM concentration on baker's yeast isomaltase, *Aspergillus niger* amyloglucosidase, green coffee bean α-galactosidase, Jack bean α-mannosidase, and Helix pomatia β-mannosidase.

Cell-based recovery of HexA in TSD-GS fibroblast

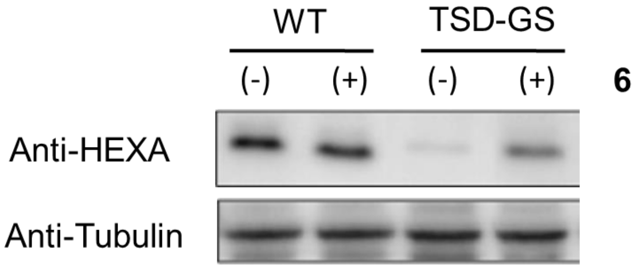

Figure S1. Immunoblot of cell lysates for chaperoning effects of compound **6** in TSD patient fibroblast. The expression levels of HexA were determined in the control and TSD-GS fibroblast by Western blotting. Tubulin was used as a loading control.

NMR spectra of new DGJNAc derivatives

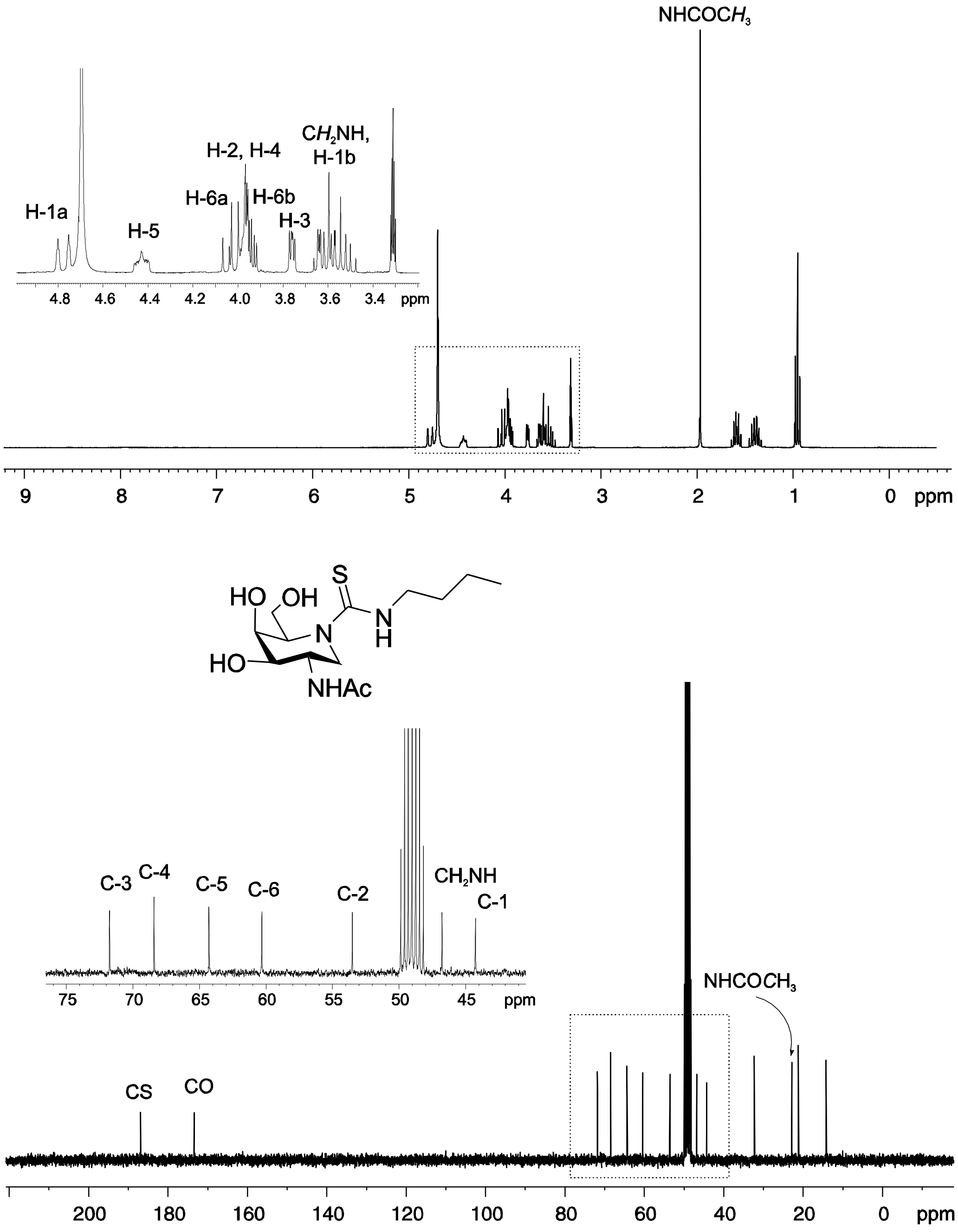

Figure S2. ^{1}H and ^{13}C NMR spectra (300 MHz, 75.5 MHz, CD_3OD) of **1**.

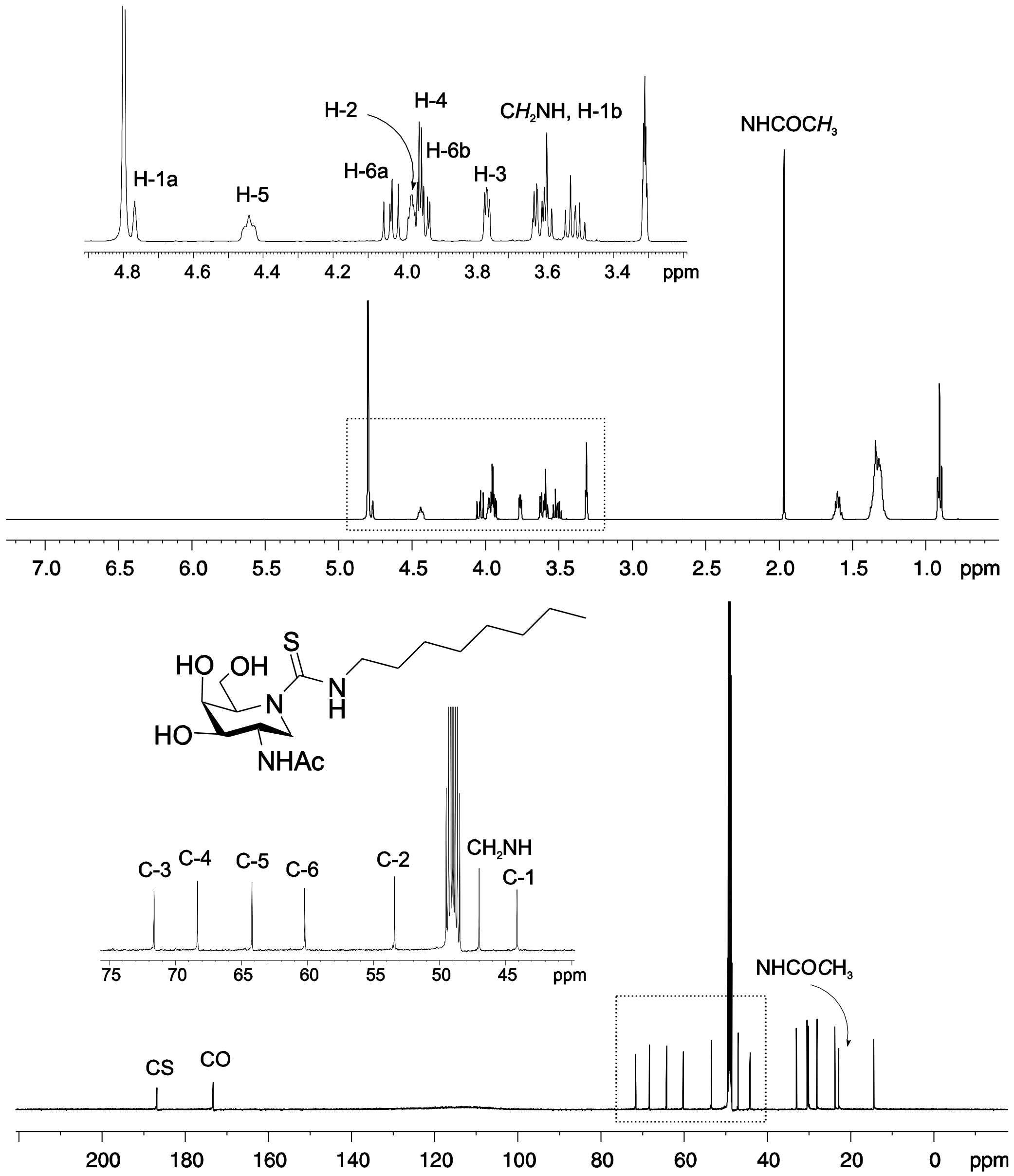

Figure S3. ^{1}H and ^{13}C NMR spectra (500 MHz, 125.7 MHz, CD_3OD) of **2**.

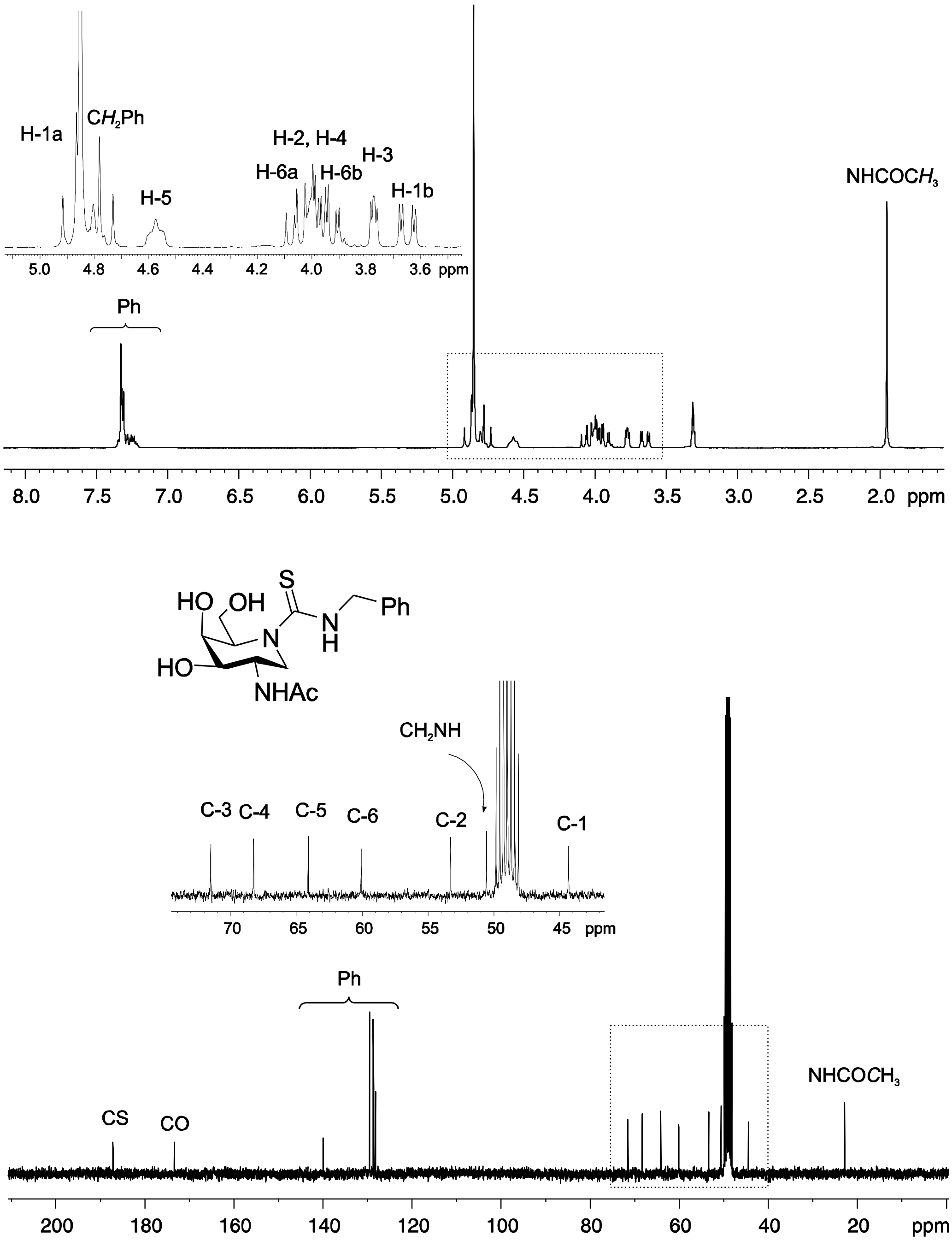

Figure S4. ^{1}H and ^{13}C NMR spectra (300 MHz, 75.5 MHz, CD_3OD) of **3**.

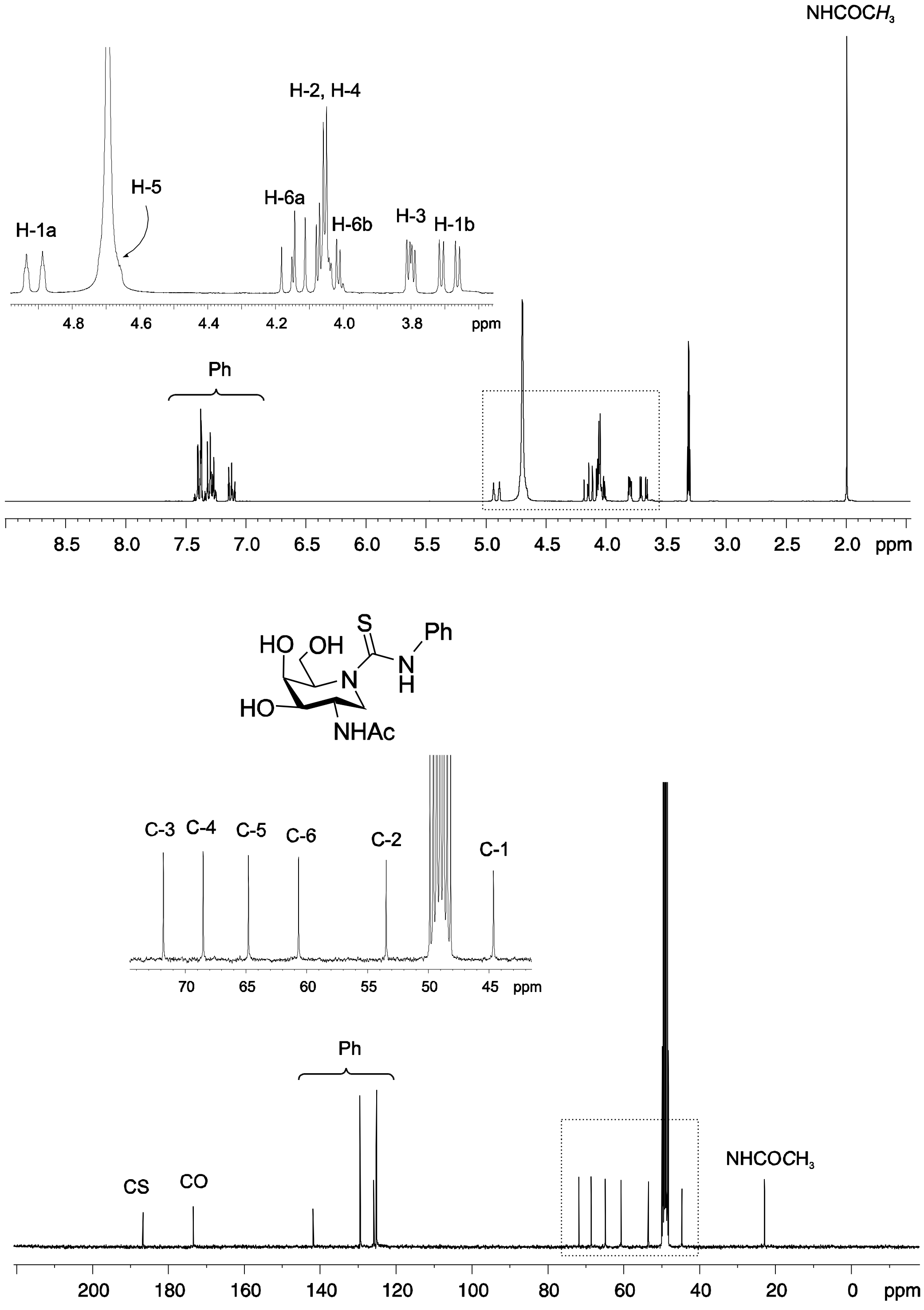

Figure S5. ^{1}H and ^{13}C NMR spectra (300 MHz, 75.5 MHz, CD_3OD) of **4**

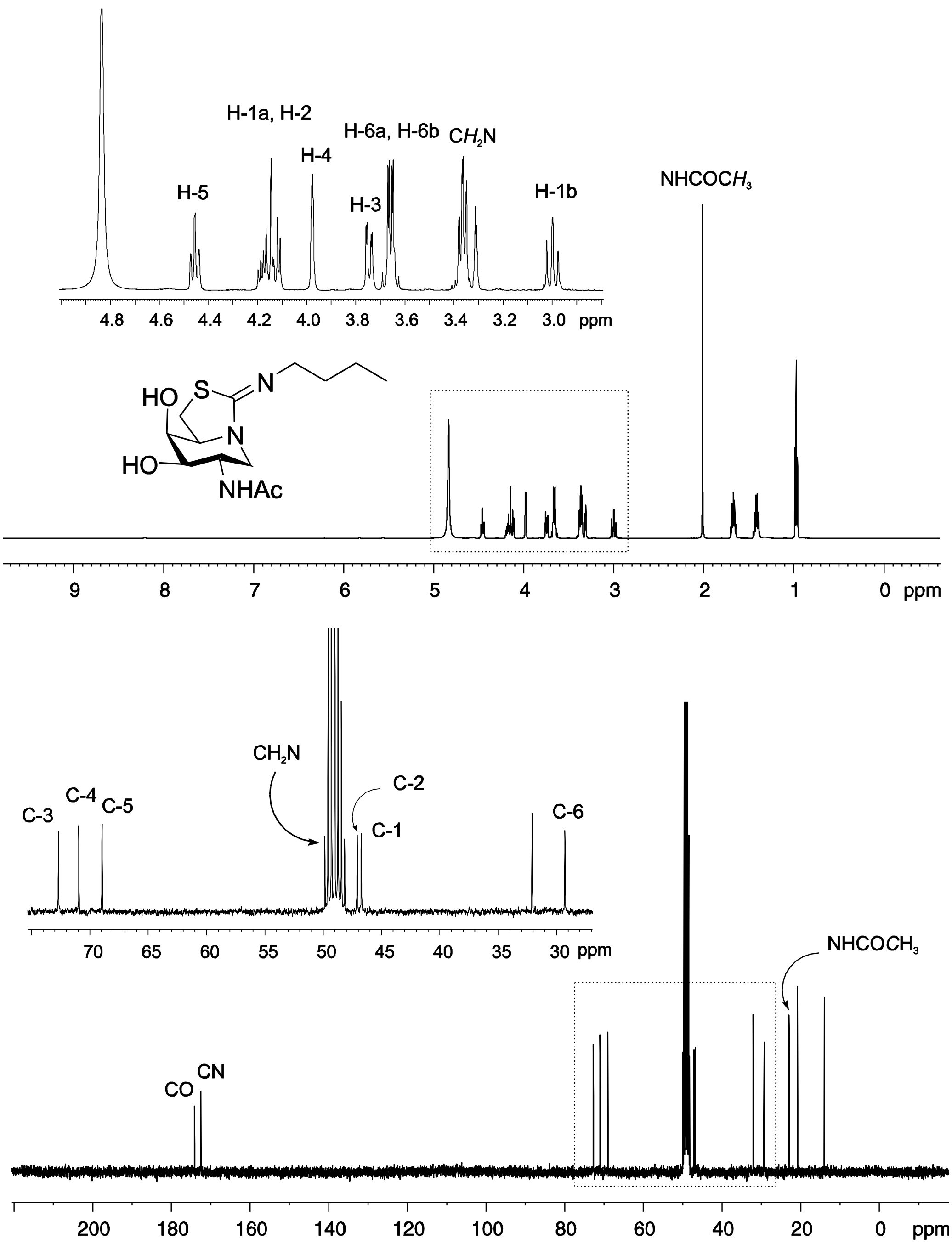

Figure S6. ^{1}H and ^{13}C NMR spectra (500 MHz, 75.5 MHz, CD_3OD) of **5**.

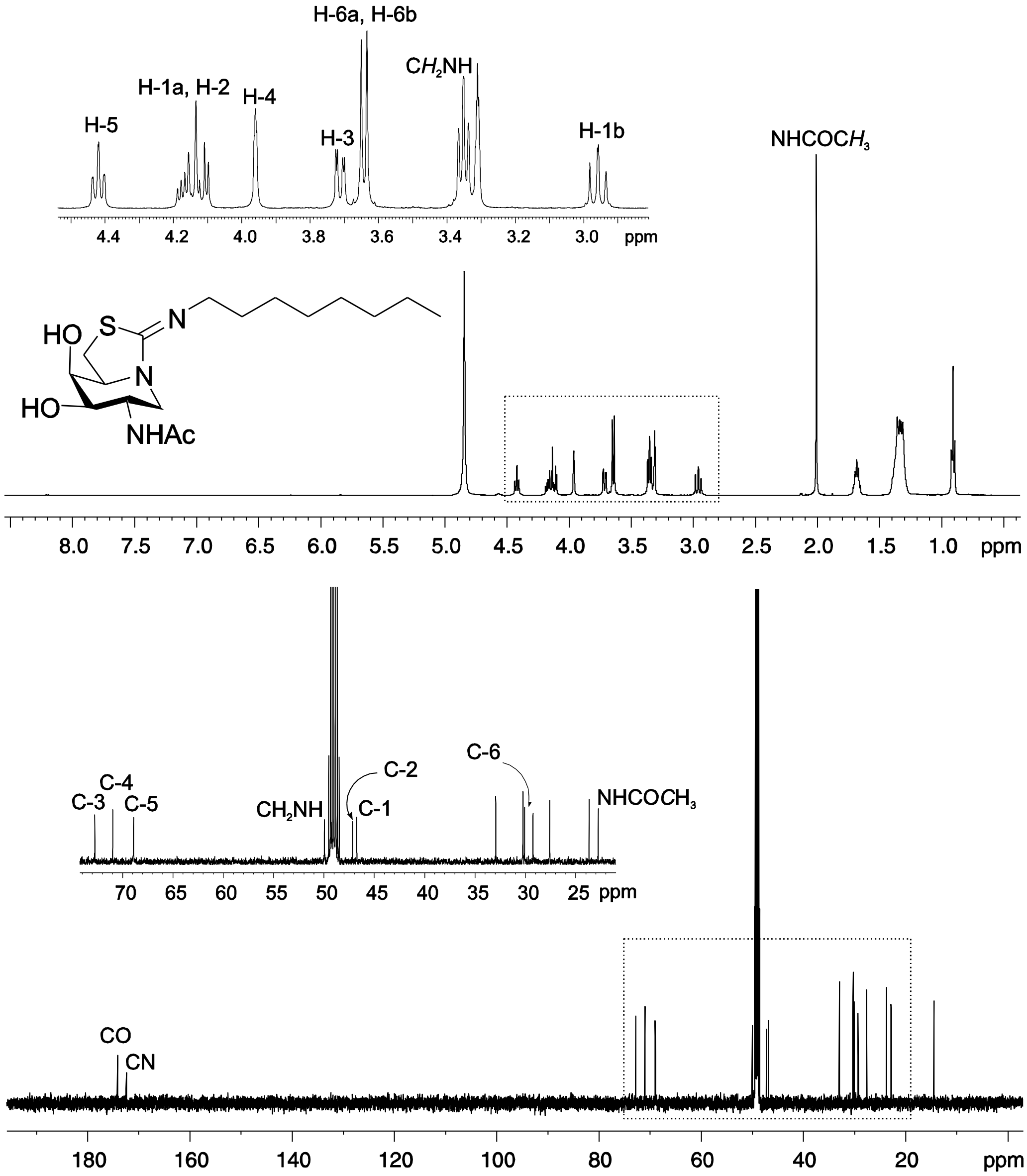

Figure S7. ^{1}H and ^{13}C NMR spectra (500 MHz, 125.7 MHz, CD_3OD) of **6**.

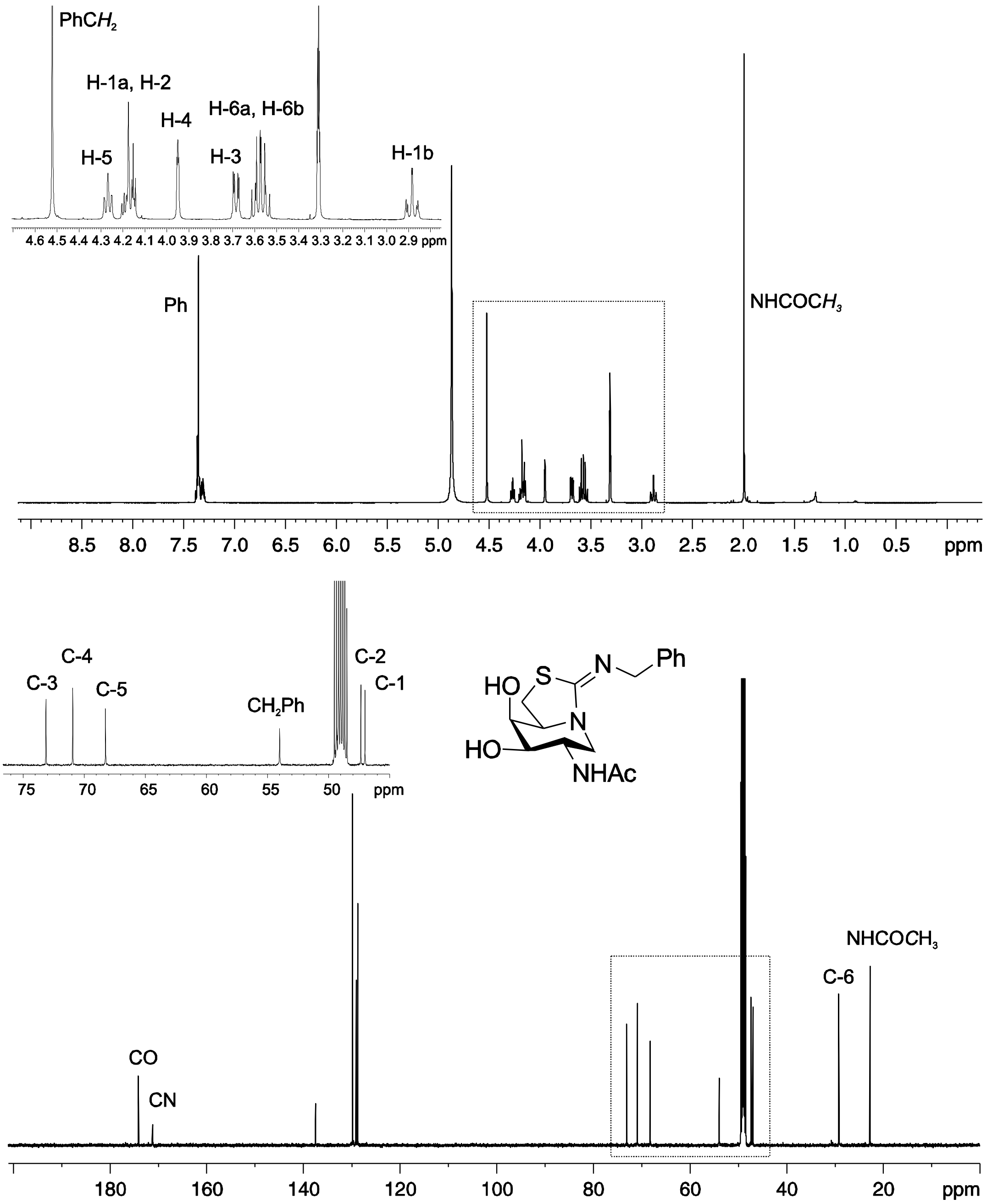

Figure S8. ^{1}H and ^{13}C NMR spectra (500 MHz, 125.7 MHz, CD_3OD) of **7**

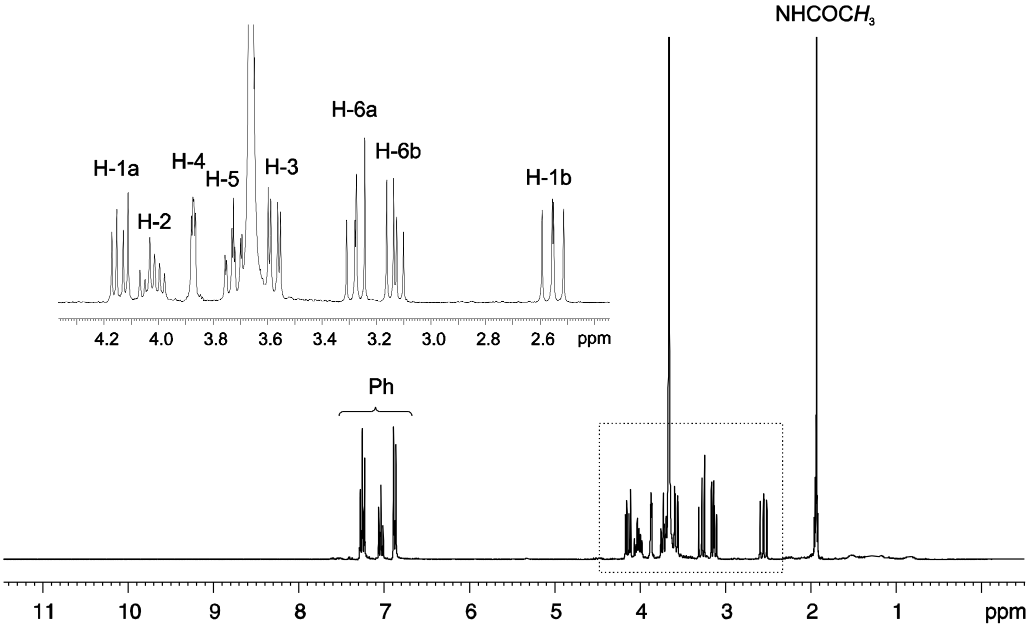

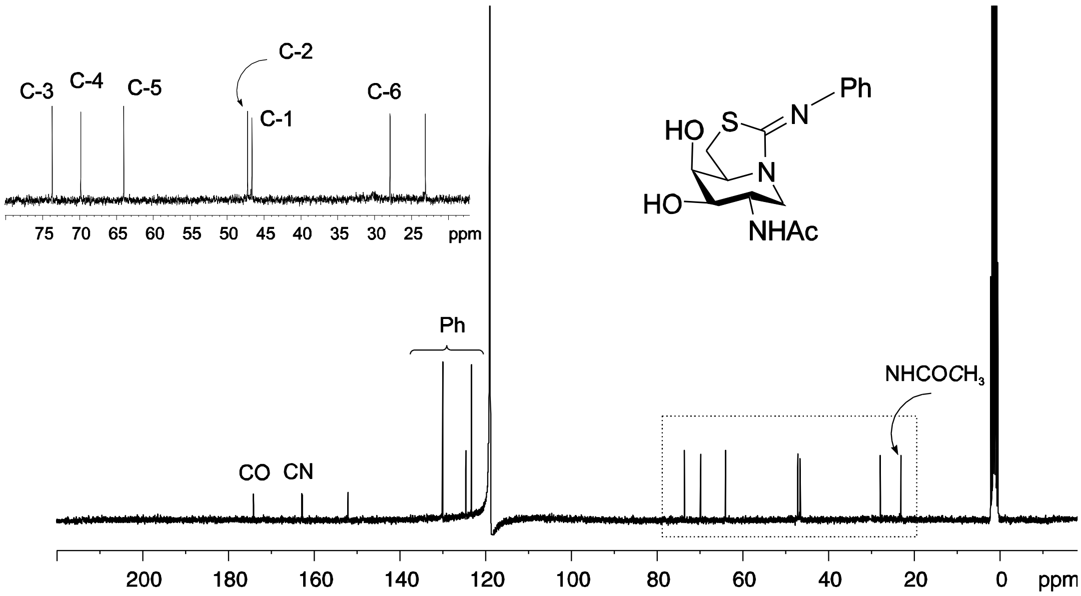

Figure S9. ^{1}H and ^{13}C NMR spectra (300 MHz, 75.5 MHz, CD_3CN-D_2O (10:1)) of **8**.

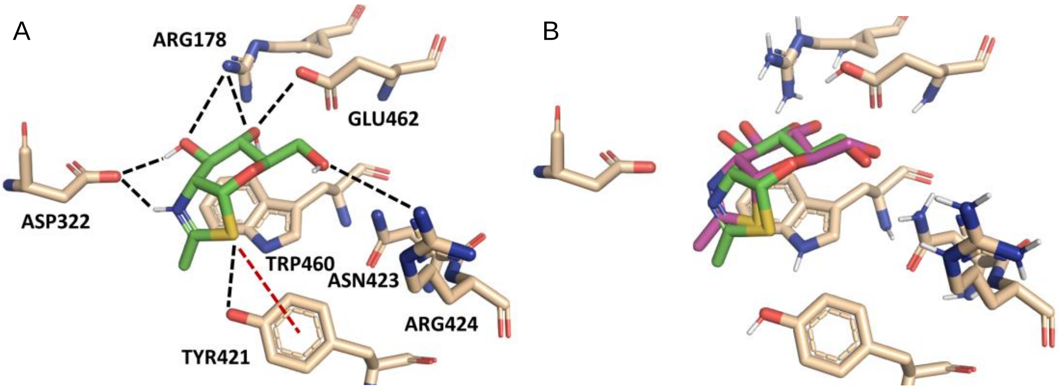

Figure S10. (A) Binding of **NAG-thiazoline** (green sticks) to HexA (tan sticks) from the crystallographic complex (PDB ID 2GK1). (B) Overlay of crystallographic **NAG-thiazoline** (green sticks) and docked **NAG-thiazoline** (magenta sticks) bound to HexA. Hydrogen atoms from ligands are not shown for clarity. Hydrogen bonds are represents as black dashed lines and sulfur-π interactions in red dashed lines.

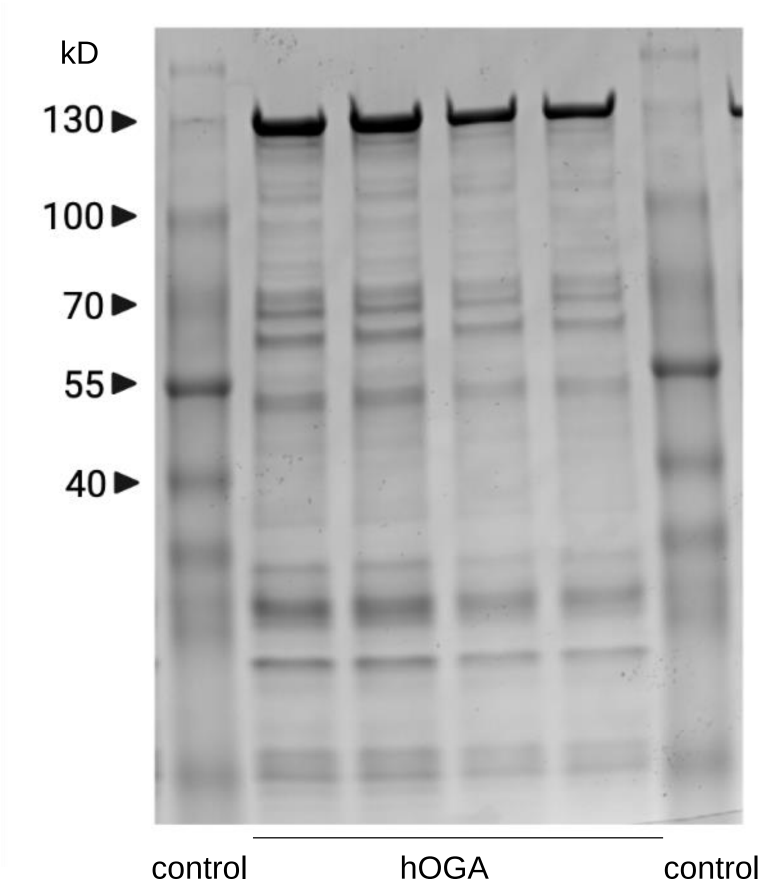

Figure S11. Immunoblot assay using anti-OGA to test the purity of hOGA. The major band at approximately 130 kD correspond to the dimeric form of the enzyme. Lower molecular mass bands arise from truncated forms of the enzyme.

References

1. Glawar AFG, Best D, Ayers BJ, Miyauchi S, Nakagawa S, Aguilar-Moncayo M, García Fernández JM, Ortiz Mellet C, Crabtree EV, Butters TD, Wilson FX, Kato A, Fleet GWJ, Efficient stereoselective synthesis of 2-acetamido-1,2-dideoxyallonojirimycin (DAJNAc) and sp^2-iminosugar conjugates: Novel hexosaminidase inhibitors with discrimination capabilities between the mature and precursor forms of the enzyme. *Chem Eur J* 2012;18:9341-9359.
